# Immune checkpoint B7x promotes immune evasion and resistance to PD-1/PD-L1 blockade in bladder cancer

**DOI:** 10.1016/j.gendis.2025.101950

**Published:** 2025-11-26

**Authors:** Marc C. Pulanco, Xiang Yu Zheng, Alexander Sankin, Deyou Zheng, Xingxing Zang

**Affiliations:** a Department of Microbiology and Immunology, Albert Einstein College of Medicine, New York 10461, USA; b Marilyn and Stanley M. Katz Institute for Immunotherapy for Cancer and Inflammatory Disorders, Albert Einstein College of Medicine, New York 10461, USA; c Department of Genetics, Albert Einstein College of Medicine, New York 10461, USA; d Department of Urology, Montefiore Medical Center and Albert Einstein College of Medicine, New York 10461, USA; e Department of Neurology, Albert Einstein College of Medicine, New York 10461, USA; f Department of Neuroscience, Albert Einstein College of Medicine, New York 10461, USA; g Department of Oncology, Albert Einstein College of Medicine, New York 10461, USA; h Department of Medicine, Albert Einstein College of Medicine, New York 10461, USA

**Keywords:** B7x (B7-H4, B7S1, or *VTCN1*), Bladder cancer, Cancer immunotherapy, Checkpoint inhibitors, Immune checkpoints, Immune evasion, Resistance mechanism, Tumor microenvironment

## Abstract

Cancer cells adopt multiple strategies to avoid detection and destruction by the immune system, including exploiting immune checkpoint pathways. B7x (B7-H4, B7S1, or *VTCN1*), a member of the B7/CD28 family, is frequently expressed in advanced bladder cancer, yet its role in bladder cancer progression and resistance to therapy remains poorly understood. Resistance to PD-1/PD-L1 immune checkpoint blockade immunotherapy significantly limits durable responses, with only 20%–25% of patients with muscle-invasive bladder cancer (MIBC) achieving long-term benefits. Here, we demonstrated that B7x mRNA and protein expression were associated with poor survival outcomes in MIBC patients and mouse models of bladder cancer, respectively. Stable expression of B7x in immune-competent bladder cancer mouse models resulted in enhanced tumor growth and splenomegaly, driven by the exclusion and suppression of tumor-infiltrating antitumor immune cells and the enrichment of pro-tumor and immunosuppressive cells. Consistently, in the IMvigor210 clinical trial, high B7x mRNA expression was correlated with poorer survival in MIBC patients treated with PD-L1 blockade. Notably, combination therapy targeting B7x alongside PD-1/PD-L1 or CTLA-4 blockade reduced tumor burden and overcame resistance to monotherapy. These findings establish B7x as a substantial driver of immune evasion in bladder cancer and highlight its potential as a therapeutic target to improve immune checkpoint blockade efficacy in MIBC.

## Introduction

Cancer cells employ various mechanisms to resist immune-mediated elimination, primarily through the manipulation of immune checkpoint pathways.^1^^,^^2^ These pathways, such as programmed cell death protein-1 (PD-1)/programmed cell death ligand-1 (PD-L1) and cytotoxic T lymphocyte antigen-4 (CTLA-4), are crucial for maintaining immune tolerance and preventing autoimmunity but can be co-opted by tumors to evade immune responses. The blockade of these checkpoints using immune checkpoint-blocking monoclonal antibodies has shown remarkable success in reactivating T-cell responses against tumors, leading to durable clinical responses in a subset of patients. However, resistance to immune checkpoint blockade (ICB) immunotherapy remains a significant challenge due to primary and acquired resistance mechanisms.^1^^,^^2^ Beyond the well-characterized PD-1/PD-L1 and CTLA-4 pathways, other immune checkpoint molecules, such as B7x, have emerged as significant players in tumor immune evasion.

B7x, also known as B7-H4, B7S1, or *VTCN1*, is an inhibitory B7 ligand of the B7/CD28 immunoglobulin superfamily^3^, ^4^, ^5^ and an immune checkpoint molecule that plays a significant role in cancer immune evasion.^6^ B7x expression is markedly elevated in various cancer types while being minimally present in normal tissues, indicating its potential as a cancer biomarker. Studies have shown that high levels of B7x protein and mRNA correlate with poor clinical outcomes,^7^, ^8^, ^9^ such as reduced overall survival and advanced cancer, suggesting its utility as a prognostic biomarker. Mechanistically, B7x contributes to immunosuppression by inhibiting T-cell activation and promoting the expansion of regulatory T cells (Tregs) and the presence of myeloid-derived suppressor cells (MDSCs)^6^^,^^10^^,^^11^ within the tumor microenvironment (TME), thereby facilitating tumor immune evasion. Additionally, B7x has been implicated in resistance to immunotherapies, such as anti-PD-1/PD-L1 and anti-CTLA-4 treatments,^11^, ^12^, ^13^, ^14^ highlighting its potential as a therapeutic target. While the role of B7x has been studied in several other cancers, the specific involvement of B7x in bladder cancer remains under investigation.

Bladder cancer is classified as non-muscle invasive bladder cancer (NMIBC) and muscle-invasive bladder cancer (MIBC), with urothelial carcinoma comprising 95% of patients.^15^ Epidemiologically, bladder cancer ranks as the fourth most common cancer in men and the eleventh in women in the United States as of 2024,^16^ with risk factors including smoking, chemical exposure, and chronic bladder irritation.^15^^,^^17^ The standard-of-care treatment for high-risk NMIBC is intravesical Bacillus Calmette-Guérin (BCG) immunotherapy, while the standard treatment for advanced MIBC is cisplatin- or carboplatin-based chemotherapy or PD-1/PD-L1 blockade immunotherapy.^15^ Despite the broad success of immune checkpoint inhibitors, such as anti-PD-1 and -PD-L1 blockade immunotherapies, their efficacy in advanced or metastatic bladder cancer is limited to a 20%–25% response rate as a first- or second-line treatment.^18^, ^19^, ^20^, ^21^, ^22^ Additionally, these immunotherapies did not show significant improvement above standard chemotherapy, with only a minority of patients responding favorably.^23^, ^24^, ^25^, ^26^ This highlights the need for alternative immunotherapeutic strategies, such as immunotherapies targeting B7x, which may expand the current cancer treatment repertoire and improve patient outcomes.

B7x is gaining recognition as an important immune checkpoint in bladder cancer, with its mRNA and protein levels correlating with adverse clinical outcomes.^27^, ^28^, ^29^, ^30^, ^31^, ^32^ While healthy bladder tissue exhibits little to no B7x protein expression, bladder cancer tissues show high levels, with 49%–76% of specimens testing positive.^27^, ^28^, ^29^ In bladder cancer, B7x protein is associated with high-grade NMIBC and MIBC, whereas B7x mRNA is associated with increased tumor-recurrence rates. B7x protein expression is mutually exclusive with PD-L1 and linked to lower densities of tumor-infiltrating CD8 T cells in bladder cancer tissues,^30^ highlighting its potential role as a distinguished immune evasion pathway. Supporting its relevance in preclinical models, canine bladder cancer shows elevated tumoral B7x levels associated with immune regulation.^31^ However, the N-butyl-N-(4-hydroxybutyl)nitrosamine (BBN)-induced spontaneous bladder cancer mouse model is reported to express B7x protein on CD11b^+^ monocytes rather than tumor cells,^29^ complicating its functional role. Furthermore, while B7x mRNA is linked to poor survival outcomes, B7x high expression combined with elevated tumor mutational burden has been associated with better responses to PD-L1 blockade immunotherapy,^32^ underscoring the need for further investigation into its function and therapeutic potential.

Here, we investigated the role of B7x in the bladder TME. Using spectral flow cytometry and RNA-sequencing (RNA-seq) approaches, we demonstrated that tumoral B7x protein suppressed and excluded tumor-infiltrating antitumor cells while promoting the infiltration of immunosuppressive cells, leading to enhanced tumor growth and poor survival in an immunogenic bladder cancer mouse model. Furthermore, we showed that high B7x expression in PD-L1 blockade-treated MIBC patients was associated with poor survival and an up-regulated epithelial–mesenchymal transition (EMT) pathway indicative of metastatic potential. Consistent with these findings, B7x^+^ bladder tumors were resistant to PD-1/PD-L1 and CTLA-4 blockade monotherapies but responded to the combination therapies with B7x blockade. Altogether, our study identifies B7x as a significant immune evasion pathway in bladder cancer and highlights the potential of targeting B7x to better current immunotherapies.

## Materials and methods

### Cell culture

The parental wild-type BBN963 (Cat# T8244) and UPPL1541 (Cat# T8245) mouse bladder cancer cell lines were obtained from Applied Biological Materials, while the MB49-luciferase mouse bladder cancer cell line was a generous gift from Dr. S. Suadicani at the Albert Einstein College of Medicine. Human breast cancer cell line SKBR3 and ovarian cancer cell line OVCAR-4 were sourced from the National Cancer Institute Developmental Therapeutics Program tumor repository. Before the start of this study, all cell lines were treated with the antimicrobial agent Primocin (InvivoGen) at a concentration of 100 μg/mL for 14 days to eliminate potential mycoplasma contamination. The BBN963, UPPL1541, and MB49 cell lines, along with their sublines, were maintained in tissue culture-treated flasks or dishes using Dulbecco’s modified Eagle medium (DMEM, Corning), supplemented with 10% heat-inactivated fetal bovine serum (FBS, R&D Systems), 1% l-glutamine (Corning), and 1% penicillin-streptomycin (100 U/mL; Corning). The SKBR3 and OVCAR-4 cell lines were cultured in Roswell Park Memorial Institute (RPMI) 1640 medium (Cat# 10-040-CV, Corning) with the addition of 10% FBS, 1% l-glutamine, and 1% penicillin-streptomycin. All cells were incubated in a humidified environment at 37 °C and 5% CO_2_. Cells were split twice weekly, and their viability was assessed using trypan blue staining with the Countess III automated cell counter (Thermo Fisher Scientific).

### Generation of stable cell lines

To establish stable B7x-expressing and B7x-negative control cell lines, we employed the murine stem cell virus (MSCV) retroviral transduction system. Full-length murine B7x was subcloned into the MSCV vector using Gibson Assembly (New England Biolabs), resulting in the MSCV-B7x construct, while the empty MSCV vector served as the control. The MSCV-B7x or MSCV-Control plasmids were cotransfected with the pVSV-g packaging plasmid into Phoenix-Ampho packaging cells, utilizing the jetPRIME transfection reagent (Polyplus) for viral production. The virus-containing supernatant was subsequently collected, sterile-filtered, and supplemented with polybrene and HEPES before being applied to target tumor cells. The cells were then subjected to spinfection by centrifugation at 1000 *g* and 37 °C for 2 hours. Four to seven days following transduction, the successfully transduced cells were purified to greater than 99% purity through flow cytometry using a BD FACSAria cell sorter.

### Mice

All animal experiments were conducted following protocols approved by the Institutional Animal Care and Use Committee (IACUC) of the Albert Einstein College of Medicine. Male wild-type C57BL/6 mice, aged 6–11 weeks, were obtained from Charles River Laboratories (CRL) or The Jackson Laboratory (JAX). All mice were housed and acclimatized in a Specific-Pathogen-Free facility for at least two weeks before experimental use. The housing conditions included a 12-hour/12-hour light/dark cycle, with temperatures between 65 and 75 °F and humidity levels at 30%–60%.

### Syngeneic tumor model

Syngeneic tumor grafts were performed using male wild-type C57BL/6 mice aged 6–11 weeks. BBN963 tumor cells, originally derived from C57BL/6 mice supplied by CRL,^33^ were engrafted into C57BL/6 mice obtained from the same source. Similarly, UPPL1541 tumor cells, originating from C57BL/6 mice from JAX, were engrafted into C57BL/6 mice from JAX.^33^ At least 24 hours before tumor-cell engraftment, the flanks and dorsal surfaces of the mice were shaved. Tumor cells were collected from culture, washed twice with 1X phosphate-buffered saline (PBS), and resuspended in PBS. Approximately 100 μL of the tumor-cell suspension was injected subcutaneously into the shaved flanks of the corresponding mice. Specifically, 3 × 10^6^ BBN963, 5 × 10^6^ UPPL1541, and 0.05 × 10^6^ MB49 cells were used for engraftment. Tumor length and width were measured every 3–4 days using calipers, and tumor volume was calculated according to the formula (L × W^2^)/2, where L is the largest diameter, and W is the shortest perpendicular diameter.^11^ Tumors were allowed to grow until they reached humane endpoints (total tumor volume exceeding 2000 mm^3^) or if the mice exhibited signs of severe illness or moribund status, at which point the animals were euthanized.

### Monoclonal antibodies

The following monoclonal antibodies (mAb) were utilized in this study: anti-mouse PD-L1 (clone 10F.9G2-CP168; Cat# CP168, Bio X Cell), with Mouse IgG1 isotype control (clone MOPC-21; Cat# BP0083, Bio X Cell); anti-mouse PD-1 (clone RMP1-14; Cat# BE0146, Bio X Cell), with Rat IgG2a isotype control (clone 2A3, Cat# BE0089, Bio X Cell); and anti-mouse CTLA-4 (clone 9D9; Cat# BE0164, Bio X Cell), with Mouse IgG2b isotype control (clone MPC-11, Cat# BE0086, Bio X Cell). The anti-B7x mAb (clone 1H3) was generated in-house using hybridoma cells cultured in Wheaton CELLine 350 or 1000 flasks (DWK Life Sciences).^34^^,^^35^ The cell compartment medium consisted of DMEM with high glucose (Cat# SH30243FS, Cytiva), supplemented with 10% ultra-low IgG FBS (Cat# 100-120-500, GeminiBio), 10% NCTC-109 (Cat# 21340039, Thermo Fisher Scientific), 1% non-essential amino acids (Cat# SH30238.01, Cytiva), and 1% penicillin-streptomycin. The medium compartment contained DMEM with high glucose (Cytiva) supplemented with 1% penicillin-streptomycin. Antibodies were purified from hybridoma supernatant using Protein G resin pulldown (Genscript). The purity, identity, and specificity of the mAbs were confirmed via SDS-PAGE and flow cytometry.

### Antibody treatment of tumor-bearing mice

Mice received subcutaneous injections of 3 × 10^6^ B7x^+^ BBN963 cells as described in the “syngeneic tumor model” section. All mAbs were administered via intraperitoneal injections. Anti-B7x, anti-PD-L1, and anti-PD-1 mAbs, along with their respective isotype controls, were administered at a dosage of 10 mg/kg (200 μg/injection). Anti-CTLA-4 mAb and its corresponding isotype control were administered at 7.5 mg/kg (150 μg/injection). In the PD-L1 blockade immunotherapy experiment, anti-B7x and isotype control treatments were given on days 1, 4, 7, 10, and 14, while anti-PD-L1 treatment was administered on days 7, 10, and 14. For the PD-1 and CTLA-4 blockade immunotherapy experiments, anti-B7x and isotype control treatments began on day 1 and continued every 3 days until day 28, with anti-PD-1 and anti-CTLA-4 treatment being introduced on day 7 and administered every 3 days until day 28. Tumor volume measurements were conducted as outlined in the “syngeneic tumor model” section.

### Tissue dissociation

To prepare tumor cell suspensions, finely minced tumor tissues were digested in 2–3 mL of a (2X) dissociation cocktail consisting of 2 mg/mL Collagenase IV (Gibco), 1 U/mL Dispase II (Sigma–Aldrich), and 10 U/mL DNase I (Roche) in FBS serum-free RPMI 1640 medium. The dissociated tissue samples were shaken and incubated at 37 °C for 30 minutes. Subsequently, the cell suspensions were passed through 100 μm cell strainers, treated with red blood cell lysis buffer (Tonbo Biosciences), and centrifuged using a discontinuous 40%–80% Percoll gradient. The interphase layer was carefully collected, washed with PBS, resuspended in RPMI medium, and used for subsequent experiments. Spleens were mechanically dissociated, treated with red blood cell lysis buffer, washed with PBS, and processed similarly for downstream applications.

### Flow cytometry

For comprehensive CD45^+^ immune cell and tumoral T-cell memory immunophenotyping, tumor and spleen cell suspensions were stained at 4 °C with LIVE/DEAD blue fixable viability dye (Invitrogen) for 20 minutes, followed by a 15-minute incubation with FcR block (Miltenyi Biotec). Subsequently, cells were stained with extracellular surface markers for 40 minutes. Intracellular staining was conducted using the eBioscience FoxP3 transcription factor staining kit (Invitrogen) according to the manufacturer’s protocol. For intracellular cytokine staining, cell suspensions were pre-stimulated for 7 hours with a PMA/ionomycin-based Cell Stimulation Cocktail (Tonbo Biosciences) before proceeding with extracellular and intracellular staining using the methods mentioned above. For cell-surface and intracellular staining, fluorophore-conjugated antibodies targeting the following mouse markers were used (Table S1): I-A/I-E (MHC-II), CD4, Ly6G, CD62L, NK1.1, CD8a, GATA-3, Ly6C, CD44, CD45, CD11b, CD206, CD11c, LAG-3, PD1, Ki-67, CD69, TCR γ/δ, TOX, RORγt, F4/80, TIM-3, Tbet, B220, Foxp3, CD3, TNFα, IL-17, IL-2, IFNγ, Granzyme B, IL-4, CD107a, Sca-1, CD122, CD127, Bcl-2, CX3CR1, KLRG1, CD27, CCR7, CD95, TCF1, CXCR3, CCR5, IRF4, Blimp1, EOMES, and Bcl-6. Data acquisition was performed on a 5-Laser Cytek Aurora spectral flow cytometer. FlowJo (BD Biosciences) software was used to analyze flow cytometry data. Uniform Manifold Approximation and Projection (UMAP) plots were produced using the UMAP plugin on FlowJo. UMAP analysis was conducted on a concatenated dataset of 16 tumor samples or 8 spleen samples, with each sample contributing an equal number of cells.

### RNA isolation

For *in vitro* RNA isolation, BBN963, UPPL1541, and MB49 cells were cultured in 5 separate tissue-culture flasks, each serving as a biological replicate, and allowed to expand for 1 week. RNA was collected using the RNeasy Plus Mini Kit (Qiagen) and stored at –80 °C. RNA quality was assessed by a Bioanalyzer RNA Pico (Agilent); samples achieving a RIN≥8.0 proceeded to sequencing. After library preparation using the SMART-seq V4 with NExtera XT kit, Admera Health performed RNA sequencing (40 million paired-end reads).

### RNA-seq data analysis

Paired-end reads were aligned to the mouse genome (mm39) using STAR (v2.7.9a).^36^ RSEM (v1.3.3)^37^ was used to quantify read counts and transcripts per million (TPM) for genes annotated in the Ensembl database (v105). Genes with a TPM ≥ 1 in at least one sample of a comparison were considered expressed and included in the downstream analyses. Differential expression analysis was computed using DESeq2 software (v1.38.3)^38^ with thresholds selected for differentially expressed gene (DEG) set at adjusted *P* < 0.05 and fold change > 1.5. Gene Set Enrichment Analysis (GSEA) was performed using the Reactome, Hallmark, Gene Ontology (GO), and Kyoto Encyclopedia of Genes and Genomes (KEGG) gene sets, using pre-ranked gene lists determined by multiplying the –log_10_(*P* value) by the log_2_(fold change).^39^ Gene clustering was performed using the STRING database (version 12.0),^40^ which provides comprehensive protein–protein association networks and functional enrichment analyses. Protein–protein interaction clustering networks for Homo sapiens were created with network edges representing confidence scores derived from various active interaction sources, including text mining, experiments, databases, co-expression, neighborhood, gene fusion, and co-occurrence. Mouse genes were mapped to their human orthologs. A minimum interaction score of 0.4 (indicating medium confidence) was used to filter the interactions. Markov Cluster Algorithm (MCL) clustering was performed with inflation parameters of 2.4 for cluster 7 and 1.7 for both cluster 5 and cluster 3. Inter-cluster edges were excluded from the visualization.

### Human datasets analysis

Differential gene expression analyses were conducted on publicly available urothelial bladder cancer datasets from The Cancer Genome Atlas (TCGA)^41^ and the IMvigor210 clinical trial.^42^ For the TCGA analysis, data levels and the specific files included in each data package are described and accessible via the National Cancer Institute (NCI) Genomic Data Commons. Cell population abundance in MIBC samples in TCGA-BLCA samples was estimated using the precalculated infiltration estimate obtained from the Timer2.0 database, which deconvolutes bulk transcriptomic data to infer the abundance of several immune cell types.^43^ In the IMvigor210 analysis, only patients categorized as complete responders (CR) or having progressive disease (PD) in the “Best Confirmed Overall Response” category were included in our analysis. Objective response rates, key gene signatures, mutation statuses, survival data, and other clinical and molecular features for each advanced bladder cancer patient were extracted by the R package IMvigor210CoreBiologies (version 1.0.0; 12-Feb-2019).^44^ Gene signature scores were calculated with the GSVA (v1.46.0) package^45^ using “ssGSEA” method^46^ on log_2_ transformed TPM values.

### Statistical analysis

All basic statistical analyses were performed using GraphPad Prism software (version 10.3.0). The normality of the data was assessed using the Shapiro–Wilk and the Kolmogorov–Smirnov tests. Parametric data were compared using an unpaired two-tailed Student’s *t*-test or one-way ANOVA with Tukey’s multiple comparisons test. Non-parametric data were analyzed using the Mann–Whitney U test (Wilcoxon rank-sum test) or the Kruskal–Wallis test. Survival data were assessed using the log-rank test. Fisher’s exact test was used for categorical data in a 2 × 2 format. The chi-squared test was applied to larger categorical datasets when the sample size assumptions were met (*n* ≥ 5). For larger contingency tables where the sample size assumptions were not met, *P* values were estimated using a chi-squared test with Monte Carlo simulation with 10,000 replicates. “Not Defined” (ND) values were excluded from the statistical analyses. Data were presented as individual values with the mean ± standard error of the mean (SEM). A *P* value or false discovery rate (FDR; *q*-value) of less than 0.05 was considered statistically significant.

## Results

### B7x correlates with poor clinical outcomes of bladder cancer patients

**Figure 1:**
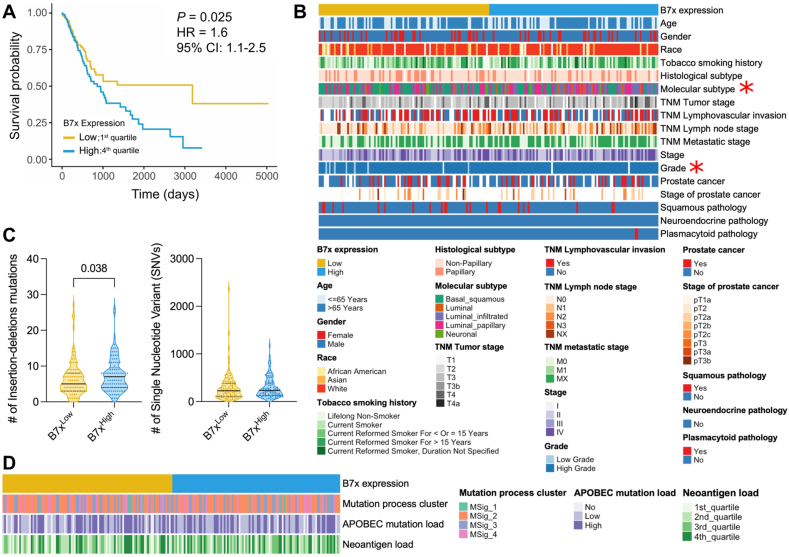
Elevated B7x mRNA expression correlates with poor prognosis in advanced or metastatic muscle-invasive bladder cancer (MIBC) patients. **(A)** Kaplan–Meier survival analysis of MIBC patients from the TCGA-BLCA dataset, stratified by B7x mRNA expression; B7x^high^ (*n* = 101 patients, 1st quartile) and B7x^low^ (*n* = 102 patients, 4th quartile). Log-rank test was used. **(B)** The heatmap illustrating demographic and clinical characteristics of the patients in (A), divided by B7x expression levels. Chi-squared or Fisher’s exact test was applied. ∗ indicates *P*-value statistical significance. **(C)** The violin plots comparing insertion-deletion and single-nucleotide variant mutations between high and low B7x expression groups. Each dot represents an individual patient. The Mann–Whitney *U* test was used; *P* values are indicated. **(D)** The heatmap illustrating mutation process cluster, APOBEC mutation load, and neoantigen load of the patients in (A), categorized by B7x expression levels. Chi-squared test was used.

To determine the clinical relevance of B7x in bladder cancer, we analyzed overall survival in a cohort of 403 patients with advanced or metastatic MIBC from TCGA. Patients were stratified based on their B7x mRNA expression (in TPM) as either high or low using three stratifying methods by either quartile values (Fig. 1A), median, or tertile (Fig. S1). High B7x expression was significantly associated with worse overall survival, with patients exhibiting a 60% higher risk of death compared to those with low B7x expression (*P* = 0.025; hazard ratio = 1.6; 95% confidence interval: 1.1–2.5; log-rank test) when evaluating with the quartile stratification (Fig. 1A). Demographic and clinical factors, such as age, gender, race, smoking history, histological subtype, TNM stage, tumor stage, pathology, prostate cancer co-presentation, neoadjuvant treatment history, noninvasive bladder history, and therapy, did not differ significantly between the B7x^high^ and B7x^low^ groups (Fig. 1B; Fig. S1B; Table S2A). However, most high-grade MIBC (51.3%; *P* = 0.014; Fisher’s exact test), luminal (83.3%), and luminal-infiltrated (75%) TCGA molecular subtypes were significantly more likely to exhibit high B7x expression (*P* = 0.0001; chi-squared test). The luminal subtype is characterized by the expression of several uroplakin and terminally differentiated urothelial umbrella cell genes, while the luminal-infiltrated is distinguished by its lower purity, presence of immune infiltrates, and the prominent expression of myofibroblast and EMT gene signatures.^47^ In the same study, the luminal-infiltrated subtype was reported to be resistant to cisplatin-based chemotherapy, while the response of the luminal subtype to chemotherapy or immunotherapy was not defined due to its novelty at the time. However, luminal micropapillary and plasmacytoid urothelial cancer subtypes are considered clinically aggressive, as they are associated with metastasis and poorer overall survival than conventional urothelial carcinoma.^48^ These results support the existing literature that links high B7x expression to high-grade tumors and poor overall survival in patients with other advanced cancers, while finding that B7x was likely to be expressed in aggressive luminal and chemoresistant luminal-infiltrated TCGA molecular subtypes of MIBC.

To evaluate whether tumor mutations were responsible for our findings, we investigated genetic mutations and neoantigen burden in our quartile cohort. Patients with high B7x expression exhibited significantly more insertion-deletion mutations (*P* = 0.038; Mann–Whitney *U* test) than those with low B7x expression (Fig. 1C). No differences were observed in the number of single-nucleotide variants between the two groups. Despite these differences in insertion-deletion mutations, there were no differences between the groups in mutational process clusters, APOBEC mutation load, and neoantigen load (Fig. 1D; Table S1B). These findings collectively suggest that B7x expression is independently driving poor overall survival in patients with advanced bladder cancer.

### Development of B7x bladder cancer mouse models

**Figure 2:**
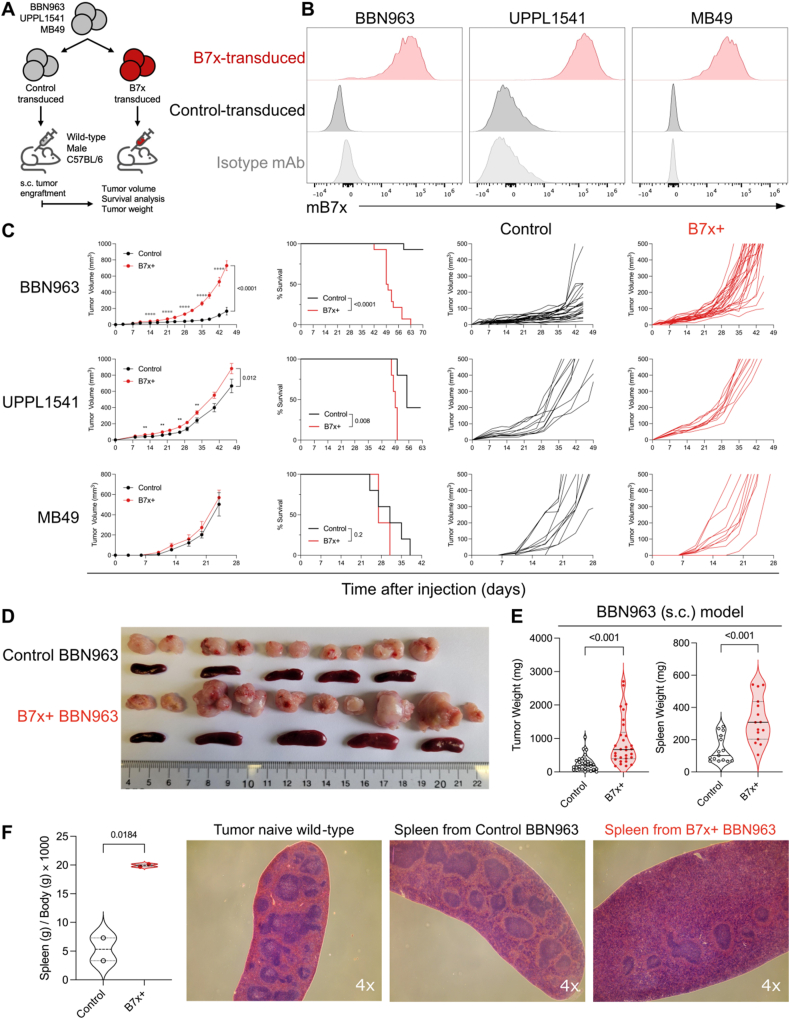
Tumoral B7x enhances bladder tumor growth in an immunocompetent host. **(A)** Schematic illustration of the generation of B7x^+^ and B7^–^ (control) sublines for BBN963, UPPL1541, and MB49 and their subcutaneous engraftment into wild-type C57BL/6 male mice. **(B)** Representative flow cytometry histogram demonstrated B7x protein expression on the tumor cell surface of B7x^+^ and B7x^–^ sublines. **(C)** Tumor growth kinetics and survival curves for B7x^+^ and B7x^–^ BBN963, UPPL1541, and MB49 sublines in immune-competent mice. Tumor volumes are shown as mean (left) and individual (right) values (BBN963, *n* = 28 tumors per group; UPPL1541 and MB49, *n* = 10 per group). Multiple unpaired *t*-tests were used (FDR *q*-value = 5%; Log-rank test). **(D)** Representative images of B7x^+^ and B7x^–^ BBN963 tumors and their associated spleens (tumors, *n* = 10 per group; spleens, *n* = 5 per group). **(E)** Comparison of tumor and spleen weights between B7x^+^ and B7x^–^ BBN963 tumor-bearing mice (tumors, *n* = 30 per group; spleens, *n* = 15 per group; two-tailed unpaired *t*-test). **(F)** Spleen-to-body weight ratio comparison and representative H&E-stained spleen sections from tumor-naïve, B7x^+^, and B7x^–^ BBN963 tumor-bearing mice (magnification, 4×; *n* = 2 spleens per group; two-tailed unpaired *t*-tests.) Error bars represent the standard error of the mean. The data represent mean ± standard error of the mean. Data in (E) and (F) are displayed as violin plots, with each dot representing an individual tumor or spleen. Corresponding *P* values are provided. H&E, hematoxylin and eosin.

To investigate the enhanced aggressiveness of B7x high/positive bladder cancer compared to B7x low/negative tumors, we developed multiple syngeneic bladder tumor mouse models. Initially, we examined the expression patterns of immune checkpoints (B7x, PD-L1, and B7-H3) on the surface of three established mouse bladder cancer cell lines: MB49, BBN963, and UPPL1541. MB49 is well-documented in bladder cancer research; however, a recent study suggests that BBN963 and UPPL1541 more accurately represent urothelial bladder cancer, while MB49 shares similarities with 3T3 mouse fibroblast cells.^33^ Flow cytometry analysis revealed that none of these cell lines naturally expressed B7x and B7-H3 proteins on their surfaces, but all displayed surface PD-L1 (Fig. S2A and S2B). Notably, BBN963 and UPPL1541 exhibited a fourfold decrease in PD-L1 expression compared to MB49. Given that human bladder cancer expressed B7x^27^, ^28^, ^29^ and some human cancer cell lines expressed endogenous B7x surface protein (Fig. S2C), we stably transduced these mouse bladder cancer cell lines to express mouse B7x surface protein, achieving expression levels comparable to those in human cell lines (Fig. 2A and B). For controls, we created B7x^–^ (control) sublines by mock-transducing the cell lines with an empty vector. We used these B7x^+^ and B7x^–^ sublines to generate syngeneic mouse models to study the role of B7x in bladder cancer.

### B7x enhances bladder cancer growth *in vivo*

To investigate the effect of B7x on tumor growth *in vivo*, we subcutaneously engrafted B7x^+^ and B7x^–^ BBN963, UPPL1541, and MB49 sublines into immune-competent wild-type male C57BL/6 mice (Fig. 2A and C). Our results demonstrated that B7x^+^ BBN963 tumors exhibited a fourfold increase in growth rate compared to the control tumors (Fig. 2C). Similarly, B7x^+^ UPPL1541 tumors showed an approximately 30% increase in tumor growth rate relative to their respective control tumors. In contrast, tumor growth was not significantly different between the B7x^+^ and control MB49 sublines. To ensure the robustness of our findings, we controlled for potential differences in mouse suppliers by sourcing animals from both JAX and CRL. We repeated the subcutaneous engraftment of B7x^+^ and control BBN963 and UPPL1541 sublines into C57BL/6 mice from different suppliers from which they originated (Fig. S2D). Consistent with our initial findings, B7x^+^ BBN963 tumors exhibited an approximately fourfold increased growth rate compared to control tumors, and B7x^+^ UPPL1541 tumors showed an approximately twofold increase growth rate compared to their control counterparts.

Given the significant tumor volume differences observed in the BBN963 bladder cancer mouse model, we extended our analysis to measure tumor and spleen weights. We studied the spleen as it is the major secondary lymphoid organ for assessing systemic immune response and alterations in immune cell distribution and activity in overall disease progression. Consistent with our tumor-volume findings, B7x^+^ BBN963 tumors were significantly heavier than the control tumors, weighing 3.53 times more after 55 days (Fig. 2D and E). Interestingly, the spleens from B7x^+^ BBN963 tumor-bearing mice were larger in size (length and width) and significantly heavier than those from control tumor-bearing mice despite no differences in body weight (Fig. 2E and F; Fig. S2E), suggesting that the B7x^+^ BBN963 tumor-bearing mice were experiencing more severe splenomegaly than their control counterparts. Hematoxylin-eosin (H&E) staining further revealed that the spleens from B7x^+^ BBN963 tumor-bearing mice exhibited a reduction in the white pulp, expansion of the red pulp, and an increase in cellularity in the red pulp (Fig. 2F), suggesting severe extramedullary hematopoiesis or splenic metastasis with a concurrent reduction in lymphoid cells. Collectively, these observations in the urothelial-like mouse bladder cancer cell lines underscore both the clinical relevance of our model system and the potent role of B7x in tumor growth.

### B7x cancer cells create an immunosuppressive tumor microenvironment

**Figure 3:**
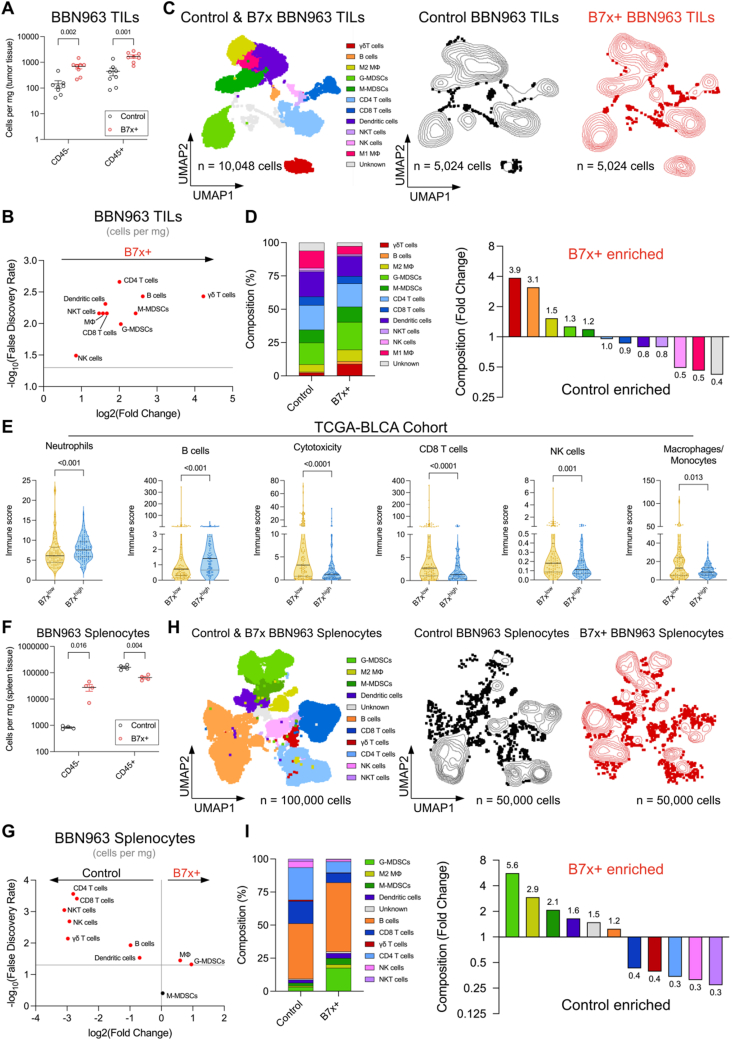
B7x^+^ bladder tumor cells exclude antitumor cells and foster an immunosuppressive tumor microenvironment. Mice engrafted with B7x^+^ and B7x^–^ BBN963 tumors, as described in Fig. 2A, were sacrificed on day 50 for tumor and spleen dissociation, followed by immunophenotyping via flow cytometry (tumors: *n* = 8 per group; spleens: *n* = 4 per group). **(A, B)** Quantification of tumor-infiltrating CD45-non-immune and CD45^+^ immune cells as cells per mg of tumor tissue, presented as mean ± standard error of the mean, with each dot representing a tumor. Multiple unpaired *t*-tests were used (FDR *q*-value = 5%). **(C, D)** Proportions of CD45^+^ immune cells quantified using unsupervised UMAP analysis are presented as stacked bar graphs alongside corresponding fold-change values. **(E)** Immune cell estimation in the B7x^high^ and B7x^low^ TCGA-BLCA cohort from Fig. 1 using MCP-Counter deconvolution analysis presented as violin plots, with each dot representing a patient. Mann–Whitney U tests were used. **(F, G)** Quantification of splenic CD45-non-immune and CD45^+^ immune cells as cells per mg of splenic tissue, shown as mean ± standard error of the mean, with each dot representing a spleen. Multiple unpaired *t*-tests were used (FDR *q*-value = 5%). **(H, I)** Proportions of splenic CD45^+^ immune cells are presented as stacked bar graphs and their corresponding fold-change values. Corresponding *P* values are indicated. TILs, tumor-infiltrating leukocytes; TCGA-BLCA, TCGA-bladder cancer; Mϕ, macrophage; UMAP, uniform manifold approximation and projection; MCP-Counter, microenvironment cell population counter.

To elucidate the immunological basis for the differential growth of B7x^+^ and B7x^–^ BBN963 tumors, we utilized high-dimensional spectral flow cytometry to compare intratumoral CD45^+^ immune-cell populations between the B7x^+^ and B7x^–^ BBN963 tumors (Fig. S3A). B7x^+^ BBN963 tumors had a higher absolute number of CD45^–^ non-immune cells and CD45^+^ immune-cell infiltrates per mg of tumor tissue (Fig. 3A). Specifically, B7x^+^ tumors showed increased absolute numbers of all CD45^+^ immune-cell types per mg of tumor tissue, with significant log_2_fold-increases in γδ T cells (4.2), B cells (2.6), monocytic (M)-MDSCs (2.5), granulocytic (G)-MDSCs (2.0), CD4 T cells (2.0), CD8 T cells (1.7), dendritic cells (1.6), macrophages (1.5), natural killer T (NKT) cells (1.5), and natural killer (NK) cells (0.94) (Fig. 3B; Fig. S3B). Despite these increases, the ratios of antitumor cells to immunosuppressive cells remained unchanged between B7x^+^ and B7x^–^ tumors (Fig. S3C). However, unsupervised UMAP dimensionality reduction analysis revealed that B7x^+^ tumors were mainly enriched in the proportions of γδ T cells, B cells, immunosuppressive CD206^+^ M2 macrophages, G-MDSCs, and M-MDSCs, while the proportions of antitumor CD8 T cells, dendritic cells, NKT cells, NK cells, and pro-inflammatory M1 macrophages were reduced (Fig. 3C and D; Fig. S3D).

To assess whether the TME of B7x^+^ mouse bladder tumors mirrors clinical observations, we used previously-computed deconvoluted data^43^^,^^49^^,^^50^ from bulk RNA-seq samples of the same B7x^high^ and B7x^low^ urothelial bladder cancer patient cohort from TCGA in Figure 1. Consistent with the comparisons between B7x^+^ and B7x^–^ BBN963 mouse bladder tumors, human B7x^high^ tumors were significantly enriched in immunosuppressive neutrophils and B cells, while showing a significant reduction in cytotoxicity, CD8 T cells, NK cells, and pro-inflammatory macrophages/monocytes compared to B7x^low^ tumors (Fig. 3E). However, there were no significant differences in the total numbers of T cells, dendritic cells, cancer-associated fibroblasts, and endothelial cells between B7x^high^ and B7x^low^ tumors (Fig. S3E). These findings demonstrate that the mouse model recapitulates the human bladder TME, further validating the data and the utility of this model.

**Figure 4:**
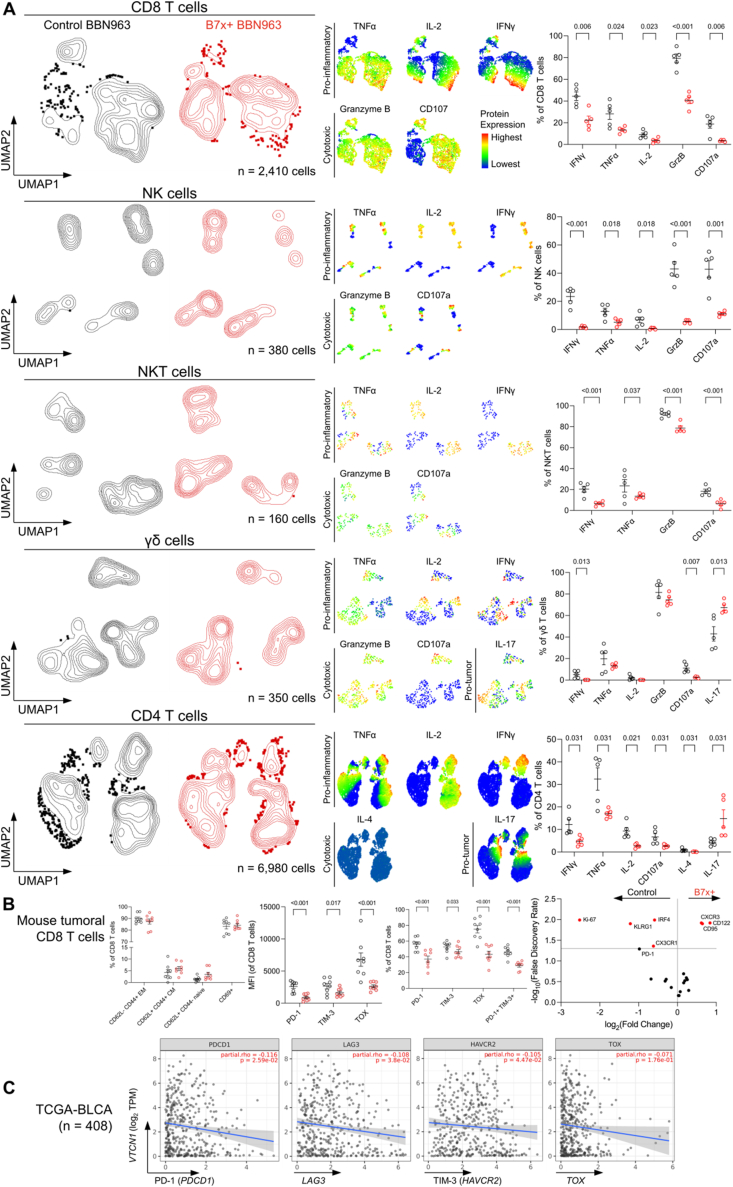
B7x^+^ bladder tumor microenvironment weakens antitumor immunity. **(A)** Cell suspensions from B7x^+^ and B7x^–^ BBN963 tumors (derived from Figure 2) were stimulated with PMA/ionomycin and intracellularly stained for Granzyme B (GrzB), CD107, IFNγ, TNFα, IL-2, IL-4, and IL-17 (*n* = 5 tumors per group). UMAP plots depict cytokine expression across immune cell populations in concatenated B7x^+^ and B7x^–^ tumors, with the proportion of cytokine ^+^ cells for each population quantified. Multiple unpaired *t*-tests were used (FDR *q*-value = 5%). **(B)** Proportions of memory and exhausted CD8 T cells, as well as the expression levels of memory and exhaustion markers, were analyzed in B7x^+^ and B7x^–^ tumors (*n* = 8 tumors per group). The volcano plot depicts fold-change in the protein expression for each memory marker in CD8 T cells. Multiple unpaired *t*-tests were used (FDR *q*-value = 5%). **(C)** Correlation analyses between B7x mRNA expression and PD-1, LAG3, TIM-3, or TOX mRNA levels were performed using the TCGA-BLCA RNA-seq dataset (*n* = 408). Best-fit linear regression lines (blue) are shown, with purity-adjusted Spearman’s Rho and corresponding *P* values (red) annotated. Data in (A) and (B) are presented as mean ± standard error of the mean, with each dot representing a tumor. Corresponding *P* values are indicated.

To gain a detailed understanding of the local and systemic effects of B7x^+^ tumors and the severe splenomegaly observed in B7x^+^ tumor-bearing mice, we conducted spectral flow cytometry analysis on the spleens of both B7x^+^ and B7x^–^ tumor-bearing mice. Consistent with the H&E staining results, which showed an expansion of red pulp and a reduction of white pulp in the spleens of B7x^+^ tumor-bearing mice (Fig. 2F), the spleens of B7x^+^ tumor-bearing mice had a higher absolute number of CD45^–^ non-immune cells per mg of spleen tissue while exhibiting a reduction in the absolute number of CD45^+^ immune cells per mg of spleen tissue compared to the controls after red blood cell lysis (Fig. 3F). Specifically, the absolute numbers of lymphoid cells, including CD4 and CD8 T cells, NKT cells, NK cells, γδ T cells, B cells, and the myeloid cells, dendritic cells, per mg of spleen tissue were significantly reduced in the spleens of B7x^+^ tumor-bearing mice compared to their B7x^–^ counterparts (Fig. 3G; Fig. S4A). Conversely, the absolute numbers of G-MDSCs and macrophages per mg of spleen tissue were significantly increased in spleens of B7x^+^ tumor-bearing mice compared to the controls. Consistent with these findings, the spleens of the B7x^+^ tumor-bearing mice exhibit a lower CD45^+^:CD45^–^ ratio, CD8 T cell:M-/G-/total MDSC ratios, and CD8 T cell:M2 macrophage ratio (Fig. S4B). UMAP analysis further confirmed that the spleens of B7x^+^ tumor-bearing mice were enriched in the proportions of immunosuppressive G-MDSCs, CD206^+^ M2 macrophages, and M-MDSCs, while the proportions of antitumor CD8 T cells, γδ T cells, CD4 T cells, NK cells, and NKT cells were reduced compared to control spleens (Fig. 4H and I; Fig. S4C). Altogether, the observed increase in lymphoid cells within the B7x^+^ TME, coupled with their concomitant decrease in the spleen, suggests enhanced lymphocyte trafficking to the B7x^+^ tumor site, possibly explaining the increase in the absolute number of CD45^+^ lymphoid cell types in the B7x^+^ tumors compared to B7x^–^ tumors.

To determine whether tumor recruitment or proliferation accounts for the increased accrual of immune cells in the B7x^+^ TME, we included the proliferation marker Ki-67 in our flow cytometry analysis. B7x^+^ tumors had a higher proportion of Ki-67^+^ CD45^–^ non-immune cells (40% increase) than B7x^–^ tumors, with no difference in Ki-67 expression (Fig. S5A). Additionally, B7x^+^ tumors exhibited a lower proportion of Ki-67^+^ B cells and CD8 T cells, consistent with decreased Ki-67 expression in these cell types. In contrast, B7x^+^ tumors had a higher proportion of Ki-67^+^ M-MDSCs, which correlated with increased Ki-67 expression in M-MDSCs. Although the proportion of Ki-67^+^ CD4 T cells, γδ T cells, NKT cells, NK cells, dendritic cells, and macrophages remained unchanged, these cells exhibited reduced Ki-67 expression in B7x^+^ tumors compared to B7x^–^ counterparts. There were no differences in the proportion of Ki-67^+^ Treg, T helper 1 (Th1), T helper 2 (Th2), and T helper 17 (Th17) CD4 T cells, but Tregs and Th1 cells showed significantly reduced Ki-67 expression in B7x^+^ tumors compared to B7x^–^ tumors (Fig. S5B). The observed increase in Tregs within the B7x^+^ TME, along with the concomitant decrease in Ki-67 expression in tumoral Tregs, suggests that B7x may be promoting the conversion of CD4 T cells into Tregs, aligning with our previous findings that B7x enhances the conversion of conventional CD4 T cells into Tregs under Treg-inducing conditions.^11^ Altogether, the data suggest that the increase in most CD45^+^ lymphoid cell types in the B7x^+^ tumors is likely due to recruitment rather than local proliferation within the TME, except for M-MDSCs.

Interestingly, spleens from B7x^+^ tumor-bearing mice had more Ki-67^+^ CD45^–^ non-immune cells with increased Ki-67 expression than their B7x^–^ counterparts (Fig. S5C). This finding suggests that the severe splenomegaly in the B7x^+^ tumor-bearing mice may be due to enhanced extramedullary hematopoiesis as a result of proliferating Ki-67^+^ CD45^–^ nucleated splenic tumor-inducible, erythroblast-like (Ter-) cells, which are present in the spleen of advanced tumor-bearing hosts.^51^ The reduction of CD45^+^ lymphoid cells and increased accumulation of Ter-cells in the spleens of B7x^+^ tumor-bearing mice relative to their B7x^–^ counterparts indicate that tumoral B7x has local and systemic effects on the proportion and number of immune cells.

### B7x-positive tumor microenvironment suppresses antitumor immunity

To determine the functionality of the antitumor immune cells in B7x^+^ tumors, we stimulated these tumor-infiltrating leukocytes and performed spectral flow cytometry to assess changes in their cytotoxicity and inflammatory phenotype (Fig. S6A). The expression of interferon-γ (IFNγ) and tumor necrosis factor-α (TNFα), as well as the proportion of IFNγ^+^ and TNFα^+^ CD8 T cells, NK cells, NKT cells, γδ T cells, and CD4 T cells, were reduced in B7x^+^ tumors compared to B7x^–^ tumors (Fig. 4A). Similarly, the expression of interleukin (IL)-2 and the proportion of IL-2^+^ CD8 T cells, NK cells, and CD4 T cells were diminished in B7x^+^ tumors. Furthermore, the expression of granzyme B (GrzB) and CD107a, along with the proportion of GrzB^+^ and CD107a^+^ CD8 T cells, NK cells, NKT cells, and γδ T cells, were lower in B7x^+^ tumors relative to B7x^–^ tumors. Unexpectedly, we observed an enrichment of IL-17 expression and a higher proportion of IL-17^+^ γδ T cells and CD4 T cells in B7x^+^ tumors compared to B7x^–^ tumors. These findings suggest that the B7x^+^ TME reduces the proportion of antitumor cells and is highly immunosuppressive, capable of suppressing the antitumor immune response while promoting pro-tumor immune cells, such as IL-17^+^ CD4 and γδ T cells.^52^, ^53^, ^54^, ^55^

To further elucidate the phenotypic state of tumoral CD8 and CD4 T cells in the B7x^+^ TME, we performed another comprehensive flow cytometry analysis focusing on their exhaustion and memory markers. Both B7x^+^ and B7x^–^ tumors predominantly comprised CD62L^–^, CD44^+^ effector memory, and CD69^+^ early activated CD8 and CD4 T cells, indicating that the T cells in BBN963 tumors are antigen-experienced, in the initial stages of activation, and retained in the TME (Fig. 4B; Fig. S6C). There was no difference in the proportion of tumoral CD62L^–^, CD44^+^ effector memory, and CD69^+^ CD8 and CD4 T cells between B7x^+^ and B7x^–^ tumors. CD8 T cells from B7x^+^ tumors exhibited reduced expression of PD-1, TIM-3, and TOX, correlating with a reduced proportion of PD-1^+^, TIM-3^+^, TOX^+^, and PD-1^+^ TIM-3^+^ CD8 T cells compared to B7x^–^ tumors (Fig. 4B). Consistent with a reduced exhaustion, inflammatory, and cytolytic phenotype, CD8 T cells from B7x^+^ tumors also showed decreased expression of the proliferation marker Ki-67, the terminal-differentiation marker KLRG1, the immune surveillance marker CX3CR1,^56^ and the activation/inflammation marker IRF4. Additionally, these CD8 T cells exhibited higher expression of the migratory/homing marker CXCR3,^56^ the survival and homeostasis marker CD122 (IL-2Rβ), and the activation-induced cell death marker CD95 (Fas). The data suggest that the CD8 T cells in the B7x^+^ TME are less cytotoxic, inflammatory, activated, proliferative, and migratory, but are undifferentiated, antigen-experienced, and tumor-homing. Thus, in bladder cancer, B7x may be stunning antigen-experienced CD8 T cells to an anergy-like early dysfunctional state,^57^ losing their effector function.

As for CD4 T cells, CD4 T cells from B7x^+^ tumors showed increased expression of TIM-3 and reduced expression of TOX, correlating with an increased proportion of TIM-3^+^ and reduced proportion of TOX^+^ cells (Fig. S6C). CD4 T cells from B7x^+^ tumors also had reduced expression of Ki-67, KLRG1, CX3CR1, and IRF4 while showing higher expression of CD122 and terminal differentiation marker Blimp1 (Fig. S6D). These findings suggest that B7x restricts most antigen-experienced CD4 T cells to a dysfunctional terminally differentiated state with reduced antitumor inflammatory effector functions, while some polarize to Th17.

To validate that B7x promotes an anergic phenotype, we correlated B7x with the exhaustion immune checkpoints and transcription factor, TOX, in MIBC patients in the TCGA database (TCGA-BLCA). B7x was significantly negatively correlated with PD-1, LAG3, and TIM-3 and exhibited a trending negative correlation with TOX in MIBC patients (Fig. 4C). To further support these findings, B7x exhibited significant negative correlations with all four exhaustion markers in TCGA patients with lung squamous cell carcinoma (LUSC), and with the three exhaustion immune checkpoints in patients with cervical and endocervical cancer (CESC) and stomach adenocarcinoma (STAD; Fig. S7A). These results suggest that B7x promotes an anergy-like early dysfunctional state in CD8 and CD4 T cells in some cancers, leading to the loss of effective antitumor immune responses.

### BBN963 bladder tumor is immunogenic and shows the immunosuppressive effects of B7x

**Figure 5:**
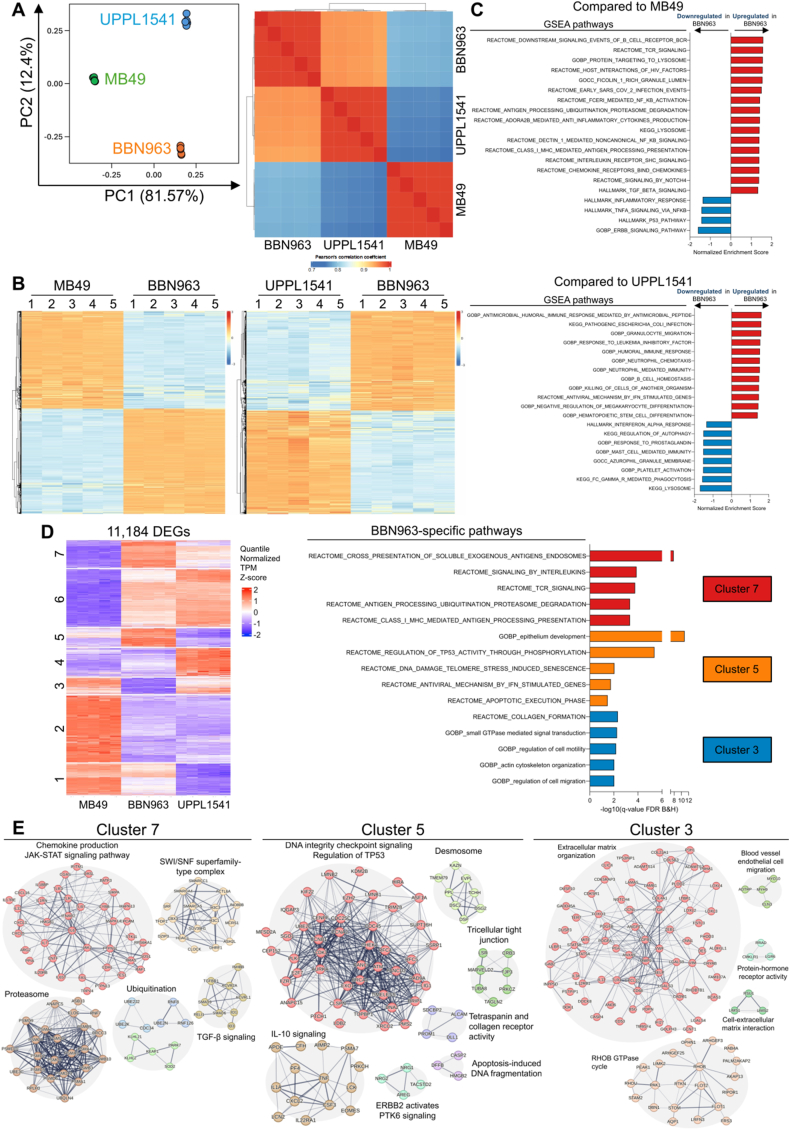
BBN963 bladder cancer cell line exhibits greater immunogenicity than UPPL1541 and MB49 cell lines. RNA-sequencing analysis of wild-type BBN963, UPPL1541, and MB49 tumor cells cultured *in vitro* (*n* = 5 replicates per cell line). **(A)** Principal component analysis (PCA) plot and Pearson’s correlation heatmap showed the clustering and relationships among the three cell lines based on all expressed genes. **(B)** The heatmaps showing the expression of differentially expressed genes (DEGs) between BBN963 and MB49 (left; 8520 DEGs: 4416 up-regulated, 4104 down-regulated in BBN963; *n* = 5) and between BBN963 and UPPL1541 (right; 4976 DEGs: 2444 up-regulated, 2532 down-regulated in BBN963; *n* = 5). **(C)** Gene set enrichment analysis (GSEA) comparing up-regulated (red) and down-regulated (blue) pathways in BBN963 versus MB49 (top) or UPPL1541 (bottom), derived from Reactome, Gene Ontology (GO), Kyoto Encyclopedia of Genes and Genomes (KEGG), and Hallmark databases (FDR < 25%). **(D)** K-means clustering of DEGs into seven clusters based on quantile-normalized transcript per million (TPM). Reactome and GO: Biological Processes (GOBP) pathway enrichment of clusters 7, 5, and 3 is highlighted. **(E)** STRING database clustering of genes within the enriched pathways from clusters 7, 5, and 3, as well as five additional immune-related pathways, showing known and predicted physical and functional associations in humans. Line thickness indicates the strength of data support (textmining, experiments, databases, co-expression, neighborhood, gene fusion, and co-occurrence). Statistical significance for DEGs and pathway analysis was determined as indicated. Corresponding *P* values are provided.

Given the significant tumor growth-enhancing effect of B7x observed in BBN963 and, to a lesser extent, UPPL1541 tumors, but not in MB49, we conducted bulk RNA-seq on BBN963, UPPL1541, and MB49 cell lines (*n* = 5) to identify immunomodulatory factors contributing to their sensitivity to B7x. Principal component analysis (PCA) revealed that the BBN963, UPPL1541, and MB49 cell lines fell into three distinct clusters, underscoring their unique molecular characteristics (Fig. 5A). Analysis of the Pearson correlation coefficients among all samples further demonstrated greater similarity between BBN963 and UPPL1541 compared to MB49. Given the pronounced tumor growth difference between B7x^+^ and B7x^–^ tumors in the BBN963 model, our focus shifted to factors specific to BBN963 cells.

Differential gene expression analysis uncovered broad transcriptomic differences between BBN963 and the other two cell lines (Fig. 5B). Gene set enrichment analysis (GSEA) highlighted the up-regulation of multiple inflammatory pathways in BBN963 compared to MB49 and UPPL1541, including those related to general and major histocompatibility complex (MHC)-I antigen processing presentation, chemokine, transforming growth factor (TGF)-β signaling, humoral response, and granulocyte/neutrophil migration, and immunity (Fig. 5C). Unexpectedly, BBN963 showed down-regulation in TNFα and IFNα signaling and response. These results suggest that BBN963 tumors may be more immunogenic and less responsive to TNFα and IFNα than MB49 and UPPL1541 at baseline.

K-means clustering analysis of all the DEGs from the BBN963 versus MB49 or UPPL1541 line identified clusters 7, 5, and 3 to be highly up-regulated, uniquely up-regulated, and uniquely down-regulated in BBN963 relative to MB49 and UPPL1541 (Fig. 5D), respectively. Function analysis by the ToppGene Suite^58^ showed that the genes of cluster 7 were enriched for antigen presentation and interleukin signaling. Cluster 5 was associated with epithelium development, TP53 activity, antiviral IFN-stimulated genes, and apoptosis. Lastly, cluster 3 genes were associated with collagen formation, GTPase signal transduction, cell motility, and migration. Consistent with the GSEA, K-means clustering and ToppGene analysis demonstrated that BBN963 is more immunogenic and possibly pro-inflammatory than MB49 and UPPL1541 at baseline.

Next, we used the STRING database to study the functional associations of genes in these clusters in more detail. Cluster 7 encompasses several pro-inflammatory chemokines and cytokines, including *Cxcl16*, *Cxcl3*, *Cxcl5*, *Il18bp*, *Il1rn*, *Il33*, and *Il6*; cell adhesion molecules *Cdh1* and *Epcam*; the apoptosis-related protein *Fas*; and immune-regulatory proteins such as *Sirpa* and *Tlr2* (Fig. 5E). Additionally, cluster 7 comprises genes associated with ubiquitination, the proteasome, chemokine production and signaling, TGF-βR1 signaling, and the SWI/SNF superfamily-type complex. Cluster 5 includes the cytokines and chemokines *Csf3*, *Cxcl2*, *Il1a*, *Pf4*, and *Tnf*, along with receptor genes *Il22ra1* and *F2rl1*. It further encompasses the lipid metabolism gene *Apoe* and the cancer stem cell marker *Prom1*. Cluster 5 features genes associated with DNA integrity, *TP53* regulation, desmosomes, tight junctions, *Erbb2* signaling, and apoptosis-related DNA fragmentation. Cluster 3 consists of the chemokines and cytokines *Tgfb1*, *Cxcl10*, and *Il34*; the cytokine receptor *Il11ra1*; the cell surface marker *Cd68*; the G protein-coupled receptor *Cmklr1*; the immunoglobulin receptor *Fcrlb*; the non-classical MHC-I molecule *H2T23*; the IFN-α inducible protein *ifi27*; the extracellular matrix protein *Loxl2*; the cell adhesion molecule *Selp*; the pattern recognition receptor *Tlr4*; and the activation ligand for the NKG2D receptor *Ulbp1*. Cluster 3 includes genes involved in extracellular matrix organization, cell-extracellular matrix interaction, the RHOB GTPase cycle, nerve growth factor signaling, and electrical signaling via rapid depolarization. Thus, the data suggest that BBN963 is immunogenic due to the expression of several pro-inflammatory chemokines and cytokines, the ubiquitination-proteasome system, SWItching/Sucrose Non-Fermentable (SWI/SNF) superfamily-type complex,^59^ cell-adhesion molecules, and proteins associated with the cellular response to DNA damage. Additionally, BBN963 has a down-regulated expression of *Tgfb1* and genes related to the organization of the extracellular matrix, suggesting that BBN963 has a reduced expression of strong immunosuppressive factors and may have a weaker physical barrier at baseline compared to UPPL1541 and MB49. Together, the data suggest that BBN963 is the most appropriate model to study the immunosuppressive effects of B7x because of its immunogenic and pro-inflammatory properties at baseline.

### B7x-positive tumors do not respond to anti-PD-1/PD-L1 immunotherapy

**Figure 6:**
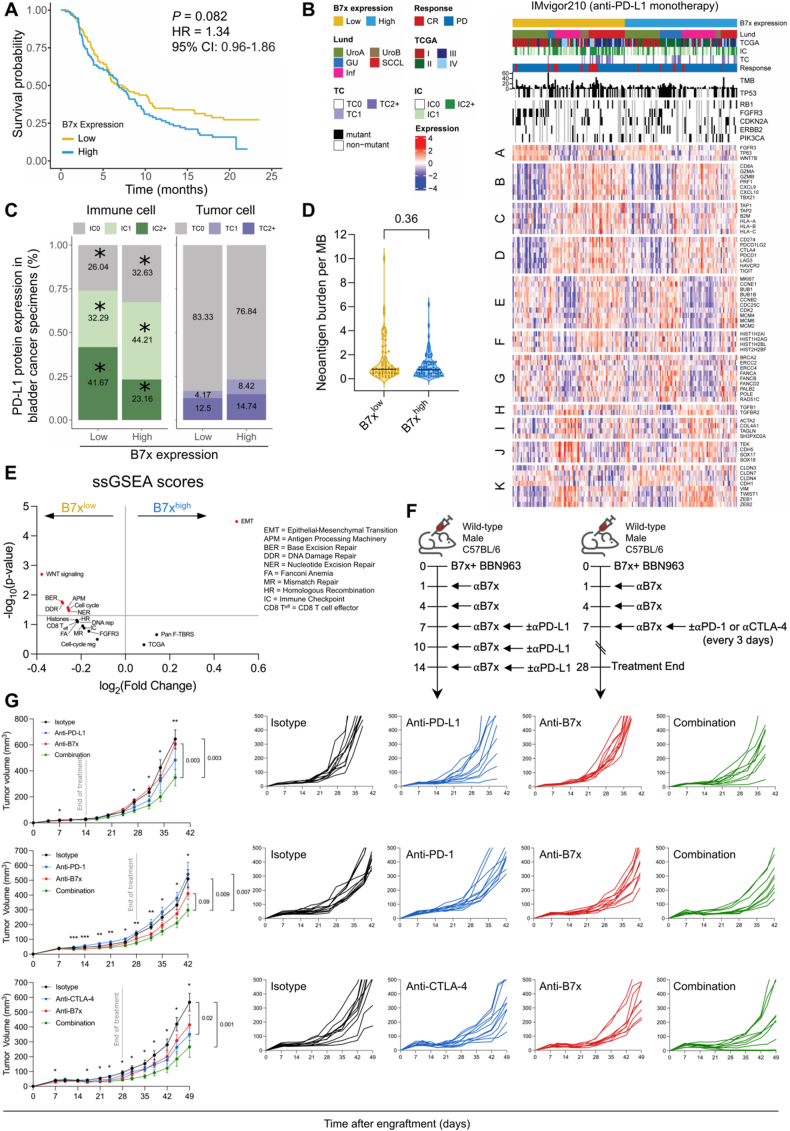
B7x mediates resistance to immune checkpoint blockade, while combination therapy with B7x blockade overcomes resistance. Analysis of complete responders and progressive disease patients with advanced or metastatic muscle-invasive bladder cancer (MIBC) treated with anti-PD-L1 blockade immunotherapy, stratified by B7x mRNA expression using a median cutoff (*n* = 96 patients per group). **(A)** The Kaplan–Meier survival curve comparing the overall survival of patients with high versus low B7x expression. Log-rank test was used. **(B)** The heatmap of clinical characteristics and bulk RNA-sequencing pathways of IMvigor210 patients stratified by B7x expression. Chi-squared or Fisher’s exact tests were applied. A, FGFR3 signaling; B, CD8 T effector cell; C, antigen presentation machinery; D, immune checkpoints; E, cell cycle; F, histones; G, DNA-damage repair; H, TGF-β signaling; I, fibroblast TGF-β response signature; J, epithelial–mesenchymal transition; K, angiogenesis. **(C)** The stacked bar graph depicting PD-L1 expression in immune cells (left) and tumor cells (right) in B7x^high^ and B7x^low^ bladder cancer samples. Chi-squared tests were applied; ∗*P* < 0.02. **(D)** The violin plot showing neoantigen burden per megabase in B7x^high^ and B7x^low^ tumors, with individual patients represented by dots. The Mann–Whitney U test was used. **(E)** Single-sample gene set enrichment analysis (ssGSEA) pathway enrichment scores for patients identified in panel A, displayed as a volcano plot with mean rank fold-change versus *P* value. Multiple Mann–Whitney U test was used (FDR *q*-value = 5%). **(F)** Experimental design schematic of B7x^+^ BBN963 tumor engraftment and treatment with B7x, PD-1, PD-L1, or CTLA-4 blockade monotherapy or combination therapies. **(G)** Mean (left) and individual (right) tumor volume tracing of B7x^+^ BBN963 tumors in immune-competent mice treated with ICB monotherapies or combination therapies. The Kruskal–Wallis test was used. Error bars represent the standard error of the mean. The data represent mean ± standard error of the mean. Corresponding *P* values are indicated throughout.

To assess the human relevance of our preclinical findings, we analyzed the overall survival of patients with high and low B7x mRNA expression in the IMvigor210 atezolizumab (PD-L1 ICB monotherapy) clinical trial, focusing on complete responders and those with progressive disease (Fig. 6; Table S3A). Consistent with our observations in the TCGA-BLCA cohort, patients with high B7x expression exhibited a trend toward lower survival probability, with a 34% higher risk of death compared to those with lower B7x expression (median cutoff; *P* = 0.082; hazard ratio = 1.34; 95% confidence interval: 0.96–1.86; log-rank test; Fig. 6A). Significant differences in key clinical variables were identified between the B7x^low^ and B7x^high^ groups (Fig. 6B; Table S3A). Notably, only 28% of complete responders were B7x^high^, compared to 72% that were B7x^low^, while a majority (53.3%) of those with progressive disease were B7x^high^ (*P* = 0.03; Fisher’s exact test). In the Lund Molecular Taxonomy subtypes, 76% of Genomically Unstable (GU) subtype tumors were B7x^high^, while B7x^high^ tumors were less frequent in the squamous cell carcinoma-like (SCCL; 31.1%) and urobasal B (UroB; 36.4%) subtypes (*P* = 0.005; chi-squared test). Within the TCGA subtypes, most subtype II tumors were B7x^high^ (71.7%) compared to 28.3% that were B7x^low^. As for mutations, those with mutated FGFR3 tended to be B7x^low^ (69.0%; *P* = 0.039; Fisher’s exact test), while those with mutated ERBB2 (HER2) were frequently B7x^high^ (80.0%; *P* = 0.015; Fisher’s exact test). Interestingly, the distribution of immune cells (IC) revealed that the IC2^+^ group had a lower proportion of B7x^high^ patients (35.5 %) compared to B7x^low^ patients (64.5%; *P* = 0.023; chi-squared test). Conversely, tumor cell (TC) analysis showed a trend where a majority of TC1 and TC2^+^ tumors were B7x^high^ (66.7% and 53.8%, respectively) compared to B7x^low^ (*P* = 0.4; chi-squared test). These findings underscore the association between high B7x expression and poor survival outcomes, non-durable responders, and reduced immune cell infiltration. Thus, the data suggest that B7x may mediate resistance in patients with MIBC treated with PD-L1 blockade immunotherapy, potentially through immune cell exclusion.

To further investigate the impact of B7x expression on antitumor inflammation and its association with molecular pathways in MIBC patients treated with PD-L1 blockade immunotherapy, we evaluated PD-L1 protein levels, neoantigen burden, and single-sample (ss)GSEA signature pathways. B7x^high^ tumors have a significant 44.4% reduction in the proportion of PD-L1^+^ IC2^+^ cells, along with significant increases of 25.3% and 36.9% in the proportions of PD-L1^+^ IC0 and IC1 cells, respectively, compared to B7x^low^ tumors (*P* = 0.023 for all comparisons; chi-squared test; Fig. 6C; Table S3B). Notably, no significant difference in PD-L1^+^ tumor cell categories (TC0, TC1, and TC2^+^) was observed between B7x^high^ and B7x^low^ tumors (*P* = 0.4; chi-squared test). These data suggest that PD-L1 expression on immune and tumor cells may not fully explain the survival difference in these patients. Although B7x^high^ tumors exhibited significantly lower tumor mutation burden per megabase (MB) compared to B7x^low^ tumors (*P* = 0.02; Mann–Whitney *U* test; Fig. S8A), there was no difference in neoantigen burden per MB between the two groups (*P* = 0.36; Mann–Whitney *U* test; Fig. 6D). This finding suggests that PD-L1 and neoantigen burden alone may not explain the survival difference observed between B7x^high^ and B7x^low^ tumors.

Further investigation into the molecular pathways using ssGSEA scores revealed that the EMT pathway was significantly up-regulated in B7x^high^ tumors, along with a trend toward up-regulation of the pan-fibroblast TGF-β response signature (Pan-FTBRS) and TCGA subtyping genes, compared to B7x^low^ tumors (Mann–Whitney *U* test; Fig. 6E; Table S3C). Conversely, gene signatures related to WNT signaling, base excision repair (BER), DNA damage response (DDR), antigen processing machinery (APM), cell cycle, and nucleotide excision repair (NER) were significantly down-regulated in B7x^high^ tumors. Additionally, there was a trend toward a lower CD8 T effector cell signature in B7x^high^ tumors, further supporting the notion that B7x^high^ tumors are less infiltrated by immune cells and exhibit a less inflammatory phenotype.

Given the potential role of tumoral B7x in mediating resistance to PD-1/PD-L1 blockade immunotherapy, we treated B7x^+^ tumor-bearing mice with anti-B7x blocking mAb alone or in combination with anti-PD-1, -PD-L1, or -CTLA-4 blocking mAbs across different treatment regimens (Fig. 6F and G). Previous studies have demonstrated that wild-type BBN963 subcutaneous tumors are responsive to PD-1/PD-L1 blockade and BCG monotherapies.^33^^,^^60^ Therefore, we aimed to determine the responsiveness of B7x^+^ BBN963 subcutaneous tumors to anti-B7x immunotherapy, with or without additional ICB immunotherapies.

Our immunotherapy studies revealed that B7x^+^ BBN963 tumors were unresponsive to PD-1/PD-L1 blockade monotherapies, aligning with our IMvigor210 human data and highlighting the need for combination therapy involving B7x blockade (Fig. 6G). However, they exhibited modest responsiveness to anti-CTLA-4 monotherapy. Notably, after the B7x plus PD-L1 blockade treatment, combination therapy significantly reduced tumor burden by 45.8% (*P* = 0.003; Kruskal–Wallis test) and 42.6% (*P* = 0.003) compared to isotype and B7x blockade monotherapies, respectively (Fig. 6G). Similarly, B7x plus PD-1 blockade combination treatment significantly reduced tumor burden compared to both isotype (41.5%; *P* = 0.009; Kruskal–Wallis test) and PD-1 (44.9%; *P* = 0.007) blockade monotherapies, with a trending reduction compared to B7x blockade monotherapy (27.3%; *P* = 0.09). In the case of B7x plus CTLA-4 blockade, anti-CTLA-4 monotherapy alone significantly reduced tumor burden by 38.1% (*P* = 0.02; Kruskal–Wallis test) compared to isotype antibody, while combination therapy further significantly reduced tumor burden by 53.0% (*P* = 0.001) compared to isotype, and showed a trending reduction compared to anti-CTLA-4 (24.0%; *P* = 0.3) and anti-B7x (35.7%; *P* = 0.2) monotherapies. These findings suggest that combining current immune checkpoint inhibitors with anti-B7x blocking mAb improves therapeutic efficacy and reduces tumor burden, underscoring the potential need for combination therapy to overcome B7x-mediated resistance in B7x^+^ tumors.

## Discussion

In this study, we discovered that B7x creates an immunosuppressive TME in bladder cancer, which excludes and suppresses antitumor immune cells, thereby accelerating tumor growth and diminishing the effectiveness of immunotherapy. An analysis of TCGA-BLCA data identified B7x as a primary driver of poor survival in MIBC patients stratified by B7x mRNA expression. Using three B7x^+^ bladder cancer mouse models, we demonstrated that B7x significantly enhanced tumor growth and worsened survival outcomes in the two urothelial models, which was further supported by spectral flow cytometry findings of suppressed CD8 T cells, NK cells, and NKT cells, alongside increased pro-tumor IL-17^+^ and immunosuppressive immune cells. RNA-seq analysis revealed that the BBN963 line was more immunogenic and pro-inflammatory than the other two models due to the up-regulation of antigen presentation and inflammatory pathways, suggesting that B7x shields immunogenic tumors from antitumor immunity. In the IMvigor210 clinical trial, B7x mRNA expression correlated with reduced survival in PD-L1 blockade-treated MIBC patients and a heightened EMT gene signature, indicating an association with metastatic and stem-like cancers. Importantly, B7x^+^ bladder tumors resisted anti-PD-1/PD-L1 and CTLA-4 ICB monotherapies but responded favorably to combination treatments with anti-B7x ICB, pointing to B7x as a viable target for improving current immunotherapies.

Previous studies have reported that B7x mRNA is associated with significantly worse overall survival in patients with urothelial bladder cancer, as analyzed using the TCGA-BLCA cohort.^29^^,^^32^ While these studies offered valuable insights, the use of markedly different analytic methods to define high and low B7x expression groups may have introduced sample size imbalances and potential biases. Another study fairly compared survival between high and low B7x expression groups using the TCGA- and Genotype-Tissue Expression (GTEx)-BLCA databases, but did not further analyze potential confounders.^31^ To build on these findings and address these considerations, we conducted a comprehensive analysis using the TCGA-BLCA cohort with evenly distributed groups based on median, tertile, and quartile stratifications. Our results consistently demonstrated that high B7x expression was associated with poor survival and was linked to high-grade tumors and luminal and luminal-infiltrated cancer subtypes, findings that align with prior studies.^29^^,^^32^ Additionally, we confirmed no significant differences in mutation or neoantigen burden between high and low B7x expression groups. These data collectively suggest that B7x is a primary driver of the observed survival differences, independent of confounding variables, further emphasizing its role in bladder cancer prognosis.

B7x protein and mRNA are elevated in human urothelial bladder cancer tissues linked to high-grade and invasive bladder cancer, tumor recurrence, and poor overall survival.^27^, ^28^, ^29^, ^30^, ^31^, ^32^ While these findings provide critical insights, there is currently no consensus on whether surface B7x on tumor cells directly promotes tumor growth or the mechanisms underlying its tumor-promoting effects in bladder cancer. A prior study using the spontaneous BBN-induced bladder cancer mouse model identified B7x expression primarily in CD11b^+^ monocytes rather than tumor cells,^29^ highlighting a potential discrepancy between human and mouse models. Since B7x is expressed on tumor cells in human bladder cancer, further research is needed to clarify its role in immune evasion and tumor progression. To address this gap, we developed three B7x^+^ mouse bladder cancer sublines expressing B7x on the tumor cell surface. By comparing B7x^+^ and B7x^–^ sublines, we found that tumoral B7x surface protein enhanced tumor growth and worsened survival in two of the three bladder cancer mouse models. To our knowledge, this is the first study to directly demonstrate the tumor-promoting role of B7x surface protein on bladder cancer cells, building on previous studies that primarily focused on using whole-body B7x-knockout mice to study the function of tumoral B7x.

In our flow cytometry analysis, we observed a notable discrepancy between the cell counts per mg and the relative proportions of immune cell subsets (UMAP analysis) between B7x^+^ and B7x^–^ bladder tumors. This discrepancy can be attributed to differences in tumor size and weight, as the larger and heavier B7x^+^ tumors exhibited higher absolute counts of all immune cell types per mg of tumor tissue. This finding indicates increased overall immune cell infiltration in B7x^+^ tumors, which is supported by the reduced expression of Ki-67 and Ki-67^+^ immune cells in the B7x^+^ tumor relative to the B7x^–^ counterpart. However, this increased infiltration did not correlate with an enhanced antitumor immune response. Instead, the relative proportion of antitumor cells was reduced, while the proportion of immunosuppressive cells was significantly higher in B7x^+^ tumors. This imbalance suggests that the B7x^+^ TME shifts toward immunosuppression, promoting tumor progression by impairing the function of antitumor cells. Thus, while B7x^+^ tumors exhibit increased immune cell infiltration overall, the altered immune cell composition highlights a more suppressive and less effective immune environment.

Our data revealed that B7x^+^ bladder tumors were populated with antitumor immune cells, including CD4 and CD8 T cells, along with NKT and NK cells, all exhibiting reduced cytotoxicity and pro-inflammatory activity. This finding, coupled with the observed enhanced tumor growth in B7x^+^ tumors, highlights the critical role of CD4 and CD8 T cells, as well as NK cells, as essential mediators of antitumor immunity in bladder cancer.^61^, ^62^, ^63^ Interestingly, a recent study demonstrated that NK cell activation and the expression of NK-activating receptors were dependent on T-cell activity in a model of allogeneic pregnancy, where B7x inhibited autoreactive CD8 T cells, reducing NK cell activation and the expression of activating receptors, which ultimately reduced fetal resorption in mice.^64^ Similarly, in our B7x^+^ bladder cancer mouse model, CD8 T cells exhibited anergy-like early dysfunction with associated suppression of NK and NKT cell activity. These findings suggest that B7x may impair the initial CD8 T cell antitumor activity, thereby hindering the subsequent activation of NK and NKT cells. This delay or weakening of the initial antitumor immune response may create an environment conducive to tumor immune evasion and progression.

Our analysis revealed that B7x^+^ tumors contained higher numbers of IL-17^+^ γδ T and CD4 T cells than B7x^–^ tumors. It is unlikely that B7x directly induces IL-17 expression in these cell types, as no studies have reported such an interaction. Instead, this increase is likely driven by factors produced by the BBN963 tumor cells. RNA-seq of BBN963 cells identified up-regulation of pathways known to promote IL-17 expression in γδ T cells and CD4 T cells, such as the IL-6^55^^,^^65^^,^^66^ and IL-1α pathways,^67^^,^^68^ distinguishing these tumors from UPPL1541 and MB49 models. This suggests that the presence of IL-17^+^ cells may result from more surviving B7x^+^ BBN963 tumor cells protected from T cell-mediated antitumor immunity. Interestingly, a recent study in pancreatic cancer has shown that IL-17/IL-17RA signaling can regulate B7x protein expression, contributing to reduced CD8 T cell infiltration and tumor development.^69^ Together, these findings propose a potential positive feedback loop in B7x^+^ tumors, where B7x protects tumor cells that produce IL-17-inducing factors, while IL-17^+^ cells help sustain tumoral B7x expression. However, other studies reported that factors such as granulocyte-macrophage colony-stimulating factor (GM-CSF), IL-10/TGF-β, progesterone, IFNγ, or IL-6 could regulate B7x protein expression, indicating that its total or cell-surface protein expression may be regulated by diverse factors across different cell types.^64^^,^^70^, ^71^, ^72^, ^73^, ^74^ Given our results, further studies are needed to elucidate the possible crosstalk between tumor and immune cells via IL-6/IL-17/IL-17RA/B7x axis and potentially other axes regulating B7x in bladder cancer progression.

Our study identified severe splenomegaly in B7x^+^ BBN963 tumor-bearing mice, characterized by an expansion of the red pulp and a significant reduction in the white pulp, which aligns with a decrease in lymphocyte populations observed in our flow cytometry data. A recent study demonstrated that ICB promotes the substantial expansion of tumor-reactive CD8 T cell clonotypes in the spleen, particularly within the white pulp, which subsequently differentiates into tumor-infiltrating antitumor clones or peripheral dysfunctional exhausted clones.^56^ The marked reduction of white pulp in B7x^+^ mice suggests a disruption of this critical process, potentially impairing the maintenance and differentiation of intermediate-exhausted CD8 T cells needed to generate effective antitumor clones. This disruption likely contributes to the enhanced tumor growth observed in B7x^+^ tumor-bearing mice by diminishing the effectiveness of tumor-reactive T-cell responses. These findings indicate that B7x^+^ tumors may exploit these mechanisms to evade immune-mediated destruction, highlighting the need for further investigation.

To understand why the tumor-promoting effects of B7x were more pronounced in the BBN963 bladder cancer mouse model compared to UPPL1541 and MB49, we performed RNA-seq analysis on the three tumor cell lines. Consistent with the previous study,^33^ our transcriptomic profiles of BBN963 and UPPL1541 were more similar to each other than to MB49, aligning with the findings that BBN963 and UPPL1541 resemble human bladder cancer and normal urothelium, whereas MB49 is more akin to fibroblasts. Our RNA-seq data revealed that BBN963 cells express numerous chemoattractants, pro-inflammatory cytokines, and pathways such as the ubiquitination-proteasome system, SWI/SNF superfamily-type complex,^59^ IL-6 pathway, TGFβR1 signaling, and DNA-damage response genes. These findings suggest that BBN963 cells have elevated DNA damage and neoantigen burden, suggesting that BBN963 is more immunogenic and visible to antitumor immunity than UPPL1541 and MB49 cells. Consistent with our findings, BBN963 tumors exhibit greater immune infiltration, effector memory T cell gene signatures, TCR clonotype sharing, and neoantigen load compared to UPPL1541 tumors, suggesting a “hot” TME.^33^ These characteristics align with human bladder cancer, known for its high tumor mutational burden and clonally expanded T cell populations.^75^, ^76^, ^77^ Thus, the immunogenic PD-L1^low^ BBN963 tumor model provides an ideal system to demonstrate that B7x functions as a critical immune-evasive pathway, suppressing antitumor immunity in bladder cancer.

A previous study using the IMvigor210 cohort reported that patients with MIBC exhibiting high B7x expression responded better to adjuvant chemotherapy and immune checkpoint blockade compared to those with low B7x expression, while also demonstrating that high B7x expression was associated with poorer survival outcomes in the TCGA-BLCA.^32^ While the conflicting findings are valuable, high B7x expression has been associated with resistance to immune checkpoint blockade in other cancers,^11^, ^12^, ^13^, ^14^ highlighting the complexity of its role. Additionally, the study included markedly different sample sizes for high and low B7x expression groups (1:4 ratio), which may have introduced sample size imbalance.^32^ To address this, we focused our analysis on complete responders and progressive disease patients, excluding partial responders and stable disease patients, to reduce variability and ensure a more robust comparison between distinct response categories. Using a median cutoff, we demonstrated that high B7x expression correlated with poor survival in MIBC patients treated with PD-L1 blockade. We further showed that B7x^+^ BBN963 bladder tumors were resistant to PD-1/PD-L1 and CTLA-4 ICB monotherapies but responded to combination therapy with B7x blockade. In contrast, wild-type BBN963 tumors were responsive to both BCG and PD-1/PD-L1 immunotherapies,^33^^,^^60^ underscoring the immunosuppressive role of B7x as a significant resistance mechanism. Recent studies have further revealed that PD-1 blockade expands tumor-infiltrating Tregs via the CD8 T cell/IL-2/ICOS axis, thereby reducing the efficacy of PD-1 immunotherapy.^78^ Building on our previous findings that B7x enhances Treg expansion and immunosuppressive activity, which limits CTLA-4 ICB immunotherapy,^11^ we propose that B7x may amplify PD-1 blockade-induced Treg expansion, further promoting their suppressive function and driving resistance to PD-1 immunotherapy. This suggests that combining B7x-targeted therapies with current immunotherapies could provide a more effective approach for treating B7x^+^ tumors.

While these findings provide valuable insights into the role of B7x in shaping the bladder TME and immunotherapy outcomes, several limitations should be considered to interpret the results thoroughly. One limitation is our use of B7x-transduced mouse bladder cancer cell lines, prompted by a previous study showing no B7x protein expression on the BBN-induced spontaneous mouse bladder cancer cells.^29^ Thus, to investigate B7x in bladder cancer, we generated three B7x-transduced mouse models, with findings in the B7x-transduced BBN963 model aligning well with human data. Additionally, our study found that B7x^+^ tumor-bearing mice had more severe splenomegaly populated by CD45-nucleated tumor-inducible splenic erythroblast-like (Ter-) cells and a reduction in the white pulp in the spleen, indicating that tumoral B7x may have local and systemic effects on the immune system, which should be studied in detail in the future. Another limitation is that although combination therapies with anti-B7x ICB exceeded the efficacy of ICB monotherapies, we did not identify the specific immune cell type driving this enhanced antitumor response, which remains an area for further exploration. Addressing these limitations could improve the relevance of our findings to diverse cancer types and inform future research; nevertheless, our study also presents significant strengths that advance the understanding of B7x in cancer biology.

This study offers several notable strengths. First, it provides substantial evidence that B7x is associated with poor survival in patients with MIBC and mouse models of bladder cancer. Second, the immunophenotyping data obtained from our mouse bladder cancer model mirrors the transcriptome-based computational immune cell infiltration results in the TCGA-BLCA cohort, enhancing the validity of our findings. Third, we demonstrated that B7x on the tumor cell surface could cultivate a highly immunosuppressive TME, correlating with dysfunctional antitumor cells in an immunogenic bladder cancer mouse model. Additionally, we found that B7x was associated with poor overall survival in MIBC patients treated with PD-L1 ICB monotherapy, supported by our observation that our B7x^+^ BBN963 bladder cancer mouse model was resistant to PD-1/PD-L1 and CTLA-4 blockade monotherapies but responded to combination therapy with B7x blockade. These findings suggest that B7x should be considered a critical actionable biomarker that promotes tumor growth, worsens survival, suppresses antitumor immunity, and mediates resistance to current immune checkpoint inhibitors.

## Conclusion

In summary, we demonstrated that tumoral B7x actively drives bladder cancer progression by suppressing and excluding antitumor immune cells, potentially contributing to resistance against current standard-of-care ICB immunotherapies. Building on our previous findings that CTLA-4 and B7x blockade reduce tumor burden in the B7x^+^ colorectal cancer mouse model, we now show that combining B7x blockade with PD-1 blockade synergistically enhances therapeutic efficacy in a preclinical model of B7x^+^ bladder cancer. This finding is significant given that PD-1/PD-L1 blockade is a standard treatment for BCG-unresponsive high-grade NMIBC and MIBC, suggesting that adding B7x blockade could improve outcomes in patients with B7x^+^ bladder cancer. Furthermore, B7x blockade may counteract the immunosuppressive effects of Treg expansion observed after PD-1 blockade, broadening its relevance to cancers treated with PD-1 inhibitors. With various anti-B7x immunotherapies currently in development and in phase I clinical trials,^79^, ^80^, ^81^ these agents hold promise for safe and effective combination therapy. Given B7x expression in a wide range of cancers with overlapping indications for PD-1/PD-L1 blockade, combining B7x blockade with existing immunotherapies represents a compelling strategy to enhance treatment efficacy and overcome resistance in patients with localized or metastatic cancers.

## CRediT authorship contribution statement

**Marc C. Pulanco:** Writing – review & editing, Writing – original draft, Visualization, Validation, Software, Resources, Methodology, Investigation, Formal analysis, Data curation, Conceptualization. **Xiang Yu Zheng:** Writing – review & editing, Visualization, Validation, Software, Resources, Methodology, Investigation, Formal analysis, Data curation. **Alexander Sankin:** Writing – review & editing, Resources, Conceptualization. **Deyou Zheng:** Writing – review & editing, Visualization, Validation, Supervision, Software, Resources, Methodology, Investigation, Formal analysis, Data curation. **Xingxing Zang:** Writing – review & editing, Supervision, Funding acquisition, Conceptualization.

## Data availability

The RNA-seq datasets are available through the NCBI Gene Expression Omnibus (GEO) under accession number GSE315244. Data supporting the findings of this study are included in the main text and supplementary materials.

## Funding

This work is supported by the 10.13039/100000002US National Institutes of Health (NIH; No. R01CA175495, R01CA262132 to Xingxing Zang), 10.13039/100000005Department of Defense (No. PC210331 to Xingxing Zang), Discovery Science Award from Sebastian Strong Foundation (US; to Xingxing Zang). Marc C. Pulanco is supported by the 10.13039/100000002US NIH (No. TL1TR002557). The Flow Cytometry Core Facility is supported by the 10.13039/100000054National Cancer Institute (NCI)-designated Montefiore Einstein Comprehensive Cancer Center (No. P30CA013330), and the Einstein-Mount Sinai Diabetes Research Center is supported by the US NIH (No. DK020541). This work utilized the Flow Cytometry Core Facility’s Cytek Aurora Multiparameter Flow Cytometer purchased with funding from a US NIH SIG grant 1S10OD026833-01.

## Conflict of interests

X. Zang is an Editorial Board member for *Genes & Diseases* and was not involved in the editorial review or the decision to publish this article. X. Zang is the inventor of patent 9447186 and 12221489, covering antibodies targeting human B7x (B7-H4, B7S1, *VTCN1*) for treating cancer. The other authors declare no conflict of interests.
