# Supplementary material for: Immune checkpoint B7x promotes immune evasion and resistance to PD-1/PD-L1 blockade in bladder cancer
Source: Genes Dis. 2025 Nov 26;13(4):101950. doi: 10.1016/j.gendis.2025.101950 (PMC13089162; doi:10.1016/j.gendis.2025.101950)
Supplement: Multimedia component 1 [file mmc1.pdf]

Supplementary Information for

Immune Checkpoint B7x Promotes Immune Evasion and Resistance to PD-1/PD-L1 Blockade in Bladder Cancer

Marc C. Pulanco, Xiang Yu Zhang, Alex Sankin, Deyou Zheng, Xingxing Zang

Corresponding author: xingxing.zang@einsteinmed.edu, deyou.zheng@einsteinmed.edu

Supplementary Figures 1-8

Supplementary Tables 1-3

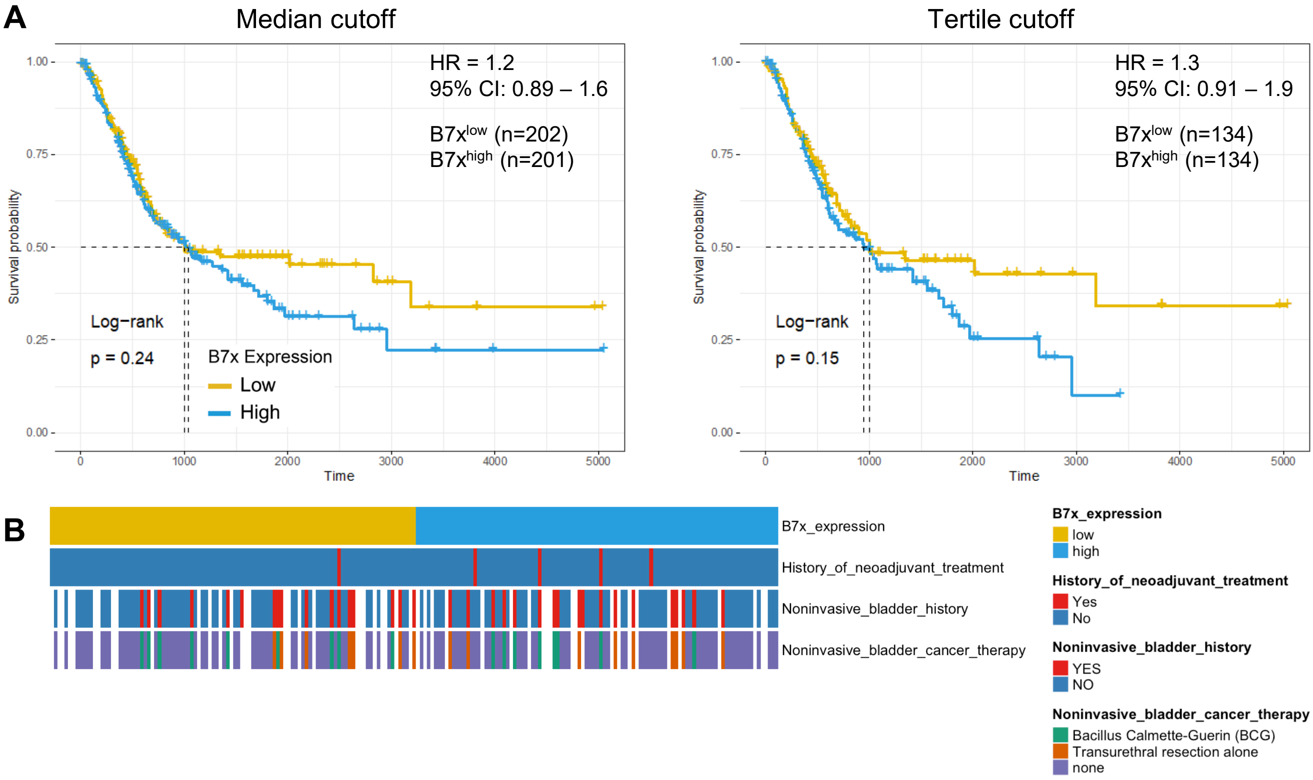
A
Median cutoff
HR = 1.2
95% CI: 0.89 – 1.6
B7x^low (n=202)
B7x^high (n=201)
Log−rank
p = 0.24
B7x Expression
Low
High
Survival probability
Time
Tertile cutoff
HR = 1.3
95% CI: 0.91 – 1.9
B7x^low (n=134)
B7x^high (n=134)
Log−rank
p = 0.15
B
B7x_expression
History_of_neoadjuvant_treatment
Noninvasive_bladder_history
Noninvasive_bladder_cancer_therapy
low
high
Yes
No
YES
NO
Bacillus Calmette-Guerin (BCG)
Transurethral resection alone
none

Figure S1

Supplementary Fig. 1. High B7x Expression Correlates with Poor Survival in MIBC Patients.

(A) Kaplan-Meier survival curves comparing overall survival of patients with advanced or metastatic MIBC stratified by B7x mRNA expression using median and tertile stratification. Log-rank test was used.

(B) Heatmap displaying clinical variables of the MIBC patients analyzed in panel (A), stratified by B7x expression levels. Chi-squared or Fisher's exact tests were applied.

Corresponding *P* values are indicated.

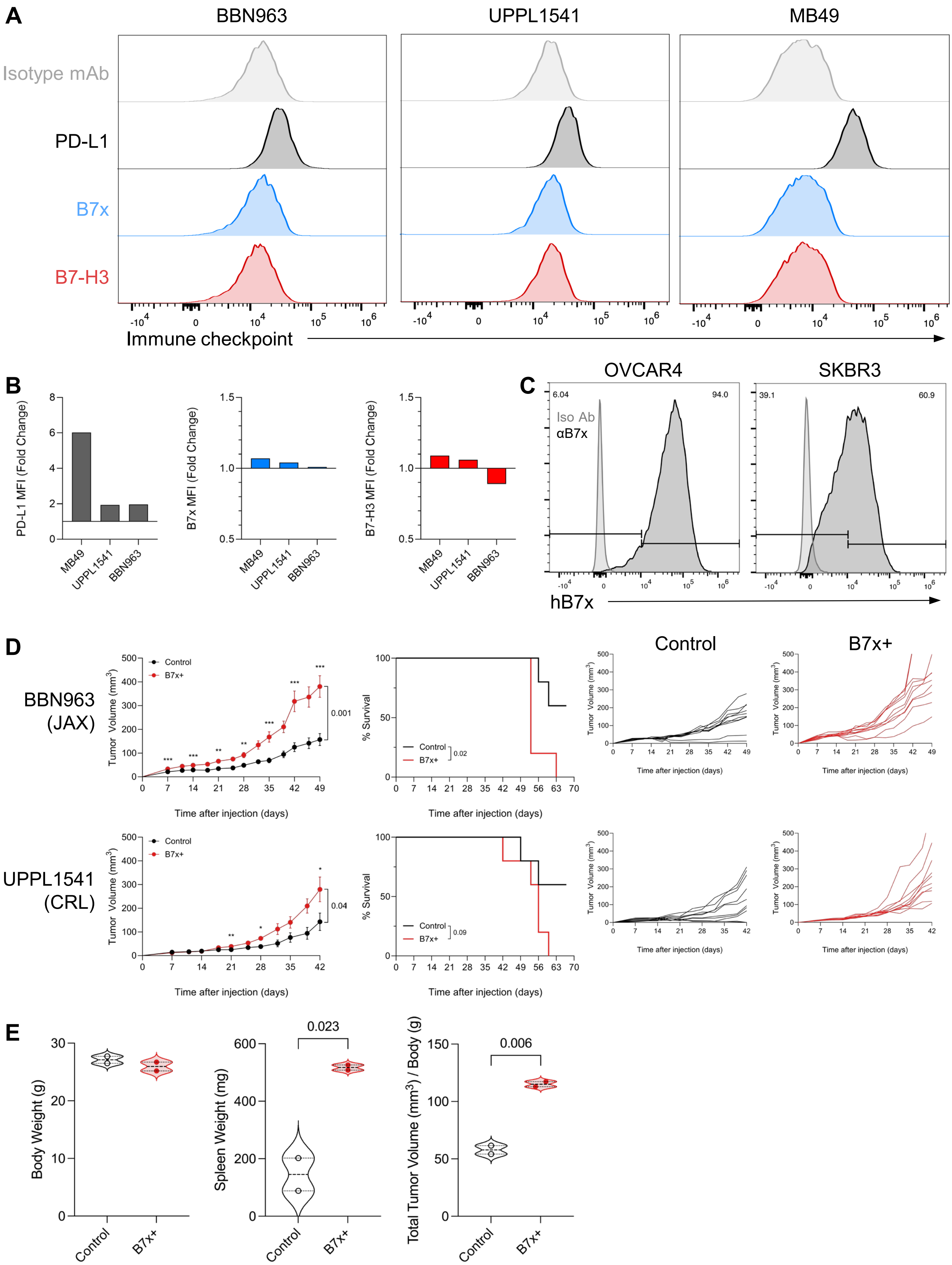

A
BBN963
UPPL1541
MB49
Isotype mAb
PD-L1
B7x
B7-H3
Immune checkpoint
B
PD-L1 MFI (Fold Change)
B7x MFI (Fold Change)
B7-H3 MFI (Fold Change)
MB49
UPPL1541
BBN963
C
OVCAR4
SKBR3
6.04
94.0
39.1
60.9
Iso Ab
αB7x
hB7x
D
BBN963 (JAX)
UPPL1541 (CRL)
Control
B7x+
Tumor Volume (mm³)
% Survival
Time after injection (days)
0.001
0.02
0.04
0.09
E
Body Weight (g)
Spleen Weight (mg)
Total Tumor Volume (mm³) / Body (g)
0.023
0.006

Figure S2

Supplementary Fig. 2. Development of the B7x+ bladder cancer mouse models.

(A) Representative flow cytometry histograms illustrating B7x, PD-L1, and B7-H3 protein expression on the tumor cell surface of parental wild-type BBN963, UPPL1541, and MB49 cell lines.

(B) Fold change in PD-L1, B7x, and B7-H3 protein expression relative to isotype control antibody for the cell lines in panel **(A)**.

(C) Representative flow cytometry histograms showing human B7x protein expression on wild-type OVCAR4 and SKBR3 human cancer cell lines.

(D) Mean (left) and individual (right) tumor volume, along with survival (middle), of B7x+ and B7x- BBN963 and UPPL1541 sublines engrafted subcutaneously into immune-competent mice from different suppliers (BBN963 and UPPL1541, n = 10 tumors per group). Multiple unpaired t-tests (FDR q-value = 5%). Log-rank test was used.

(E) Body weight, spleen weight, and total tumor volume/body weight ratios of B7x+ and B7x- (control) BBN963 tumor-bearing mice. Total tumor volume represents the combined volume of left- and right-flank tumors in each mouse. Two-tailed unpaired t-test. Data are shown as violin plots, with each dot representing a tumor. Corresponding *P* values are indicated.

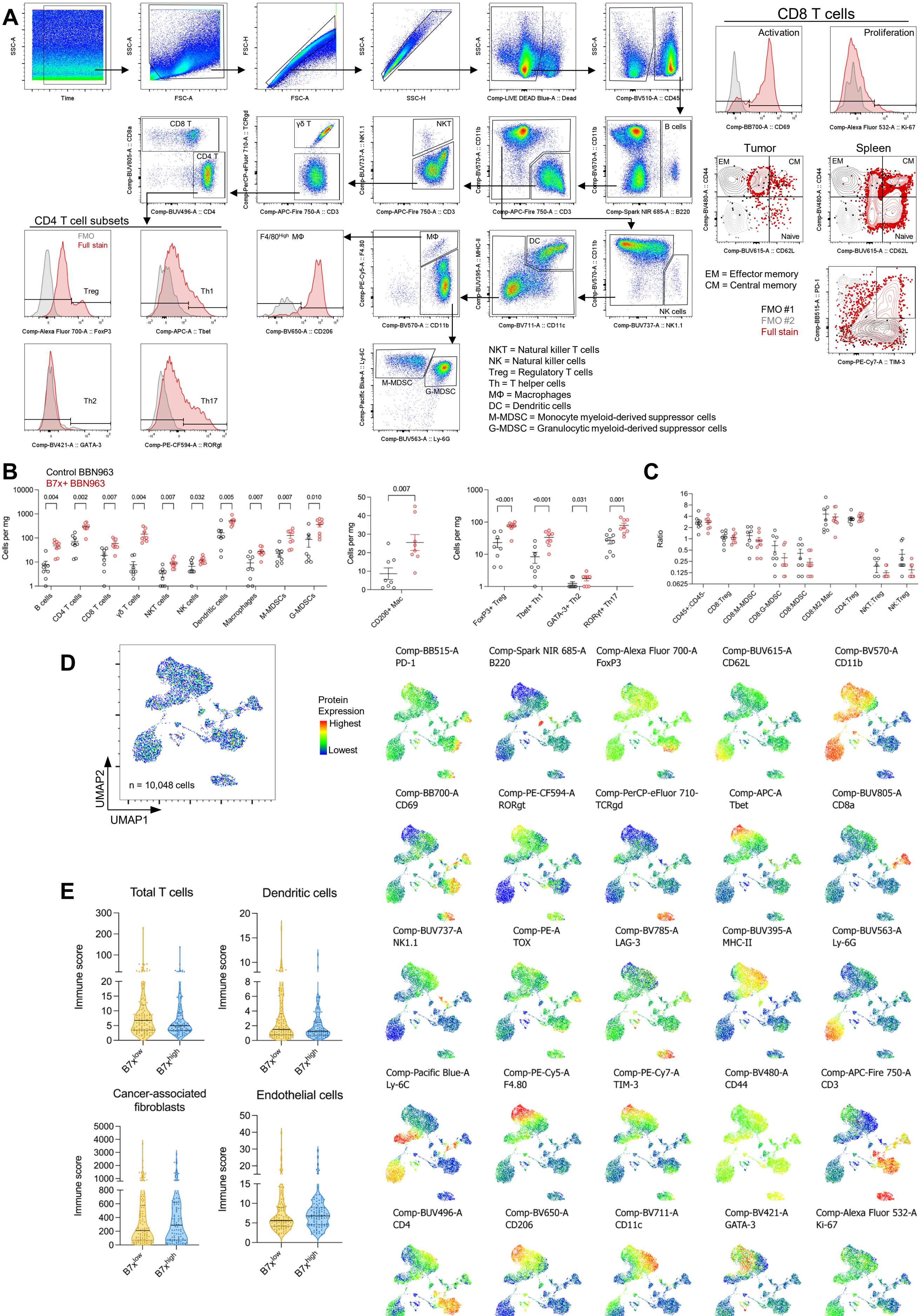
A
SSC-A
Time
FSC-A
FSC-H
SSC-H
Comp-LIVE DEAD Blue-A :: Dead
Comp-BV510-A :: CD45
CD8 T cells
Activation
Proliferation
Comp-BB700-A :: CD69
Comp-Alexa Fluor 532-A :: Ki-67
Tumor
Spleen
EM
CM
Naive
Comp-BV480-A :: CD44
Comp-BUV615-A :: CD62L
EM = Effector memory
CM = Central memory
FMO #1
FMO #2
Full stain
Comp-BB515-A :: PD-1
Comp-PE-Cy7-A :: TIM-3
CD8 T
CD4 T
Comp-BUV805-A :: CD8a
Comp-BUV496-A :: CD4
γδ T
Comp-PerCP-eFluor 710-A :: TCRgd
Comp-APC-Fire 750-A :: CD3
NKT
Comp-BUV737-A :: NK1.1
Comp-BV570-A :: CD11b
B cells
Comp-Spark NIR 685-A :: B220
CD4 T cell subsets
FMO
Full stain
Treg
Comp-Alexa Fluor 700-A :: FoxP3
Th1
Comp-APC-A :: Tbet
F4/80High MΦ
Comp-BV650-A :: CD206
MΦ
Comp-PE-Cy5-A :: F4.80
DC
Comp-BUV395-A :: MHC-II
Comp-BV711-A :: CD11c
NK cells
Comp-BUV737-A :: NK1.1
Th2
Comp-BV421-A :: GATA-3
Th17
Comp-PE-CF594-A :: RORgt
M-MDSC
G-MDSC
Comp-Pacific Blue-A :: Ly-6C
Comp-BUV563-A :: Ly-6G
NKT = Natural killer T cells
NK = Natural killer cells
Treg = Regulatory T cells
Th = T helper cells
MΦ = Macrophages
DC = Dendritic cells
M-MDSC = Monocyte myeloid-derived suppressor cells
G-MDSC = Granulocytic myeloid-derived suppressor cells
B
Control BBN963
B7x+ BBN963
Cells per mg
0.004
0.002
0.007
0.004
0.007
0.032
0.005
0.007
0.007
0.010
B cells
CD4 T cells
CD8 T cells
γδ T cells
NKT cells
NK cells
Dendritic cells
Macrophages
M-MDSCs
G-MDSCs
0.007
CD206+ Mac
<0.001
<0.001
0.031
0.001
FoxP3+ Treg
Tbet+ Th1
GATA-3+ Th2
RORγt+ Th17
C
Ratio
CD45+:CD45-
CD8:Treg
CD8:M-MDSC
CD8:G-MDSC
CD8:MDSC
CD8:M2 Mac
CD4:Treg
NKT:Treg
NK:Treg
D
n = 10,048 cells
UMAP1
UMAP2
Protein Expression
Highest
Lowest
Comp-BB515-A PD-1
Comp-Spark NIR 685-A B220
Comp-Alexa Fluor 700-A FoxP3
Comp-BUV615-A CD62L
Comp-BV570-A CD11b
Comp-BB700-A CD69
Comp-PE-CF594-A RORgt
Comp-PerCP-eFluor 710-A TCRgd
Comp-APC-A Tbet
Comp-BUV805-A CD8a
Comp-BUV737-A NK1.1
Comp-PE-A TOX
Comp-BV785-A LAG-3
Comp-BUV395-A MHC-II
Comp-BUV563-A Ly-6G
Comp-Pacific Blue-A Ly-6C
Comp-PE-Cy5-A F4.80
Comp-PE-Cy7-A TIM-3
Comp-BV480-A CD44
Comp-APC-Fire 750-A CD3
Comp-BUV496-A CD4
Comp-BV650-A CD206
Comp-BV711-A CD11c
Comp-BV421-A GATA-3
Comp-Alexa Fluor 532-A Ki-67
E
Total T cells
Dendritic cells
Cancer-associated fibroblasts
Endothelial cells
Immune score
B7x low
B7x high

Figure S3

Supplementary Fig. 3. B7x cancer cells shift the tumor microenvironment towards immunosuppressive.

(A) Gating strategy for comprehensive immunophenotyping of tumor-infiltrating lymphoid and myeloid cells in B7x+ and B7x- (control) BBN963 tumors.

(B) Quantification of immune cell populations per mg of tumor tissue in B7x+ and B7x- tumors (n = 8 tumors per group). Multiple unpaired t-tests (FDR q-value = 5%).

(C) Ratios of antitumor to immunosuppressive immune cell populations in tumors from panel **(A)**. Multiple unpaired t-tests (FDR q-value = 5%).

(D) UMAP plots illustrating protein expression of lineage and phenotypic markers in tumor-infiltrating immune cells from concatenated B7x+ and B7x- tumors.

(E) Violin plots depict the quantification of immune and non-immune cell populations in the $B7x^{high}$ and $B7x^{low}$ expressing TCGA-BLCA cohort from Fig. 1, analyzed using MCP-Counter deconvolution. Each dot represents a patient. Corresponding *P* values are indicated. Mann-Whitney U test.

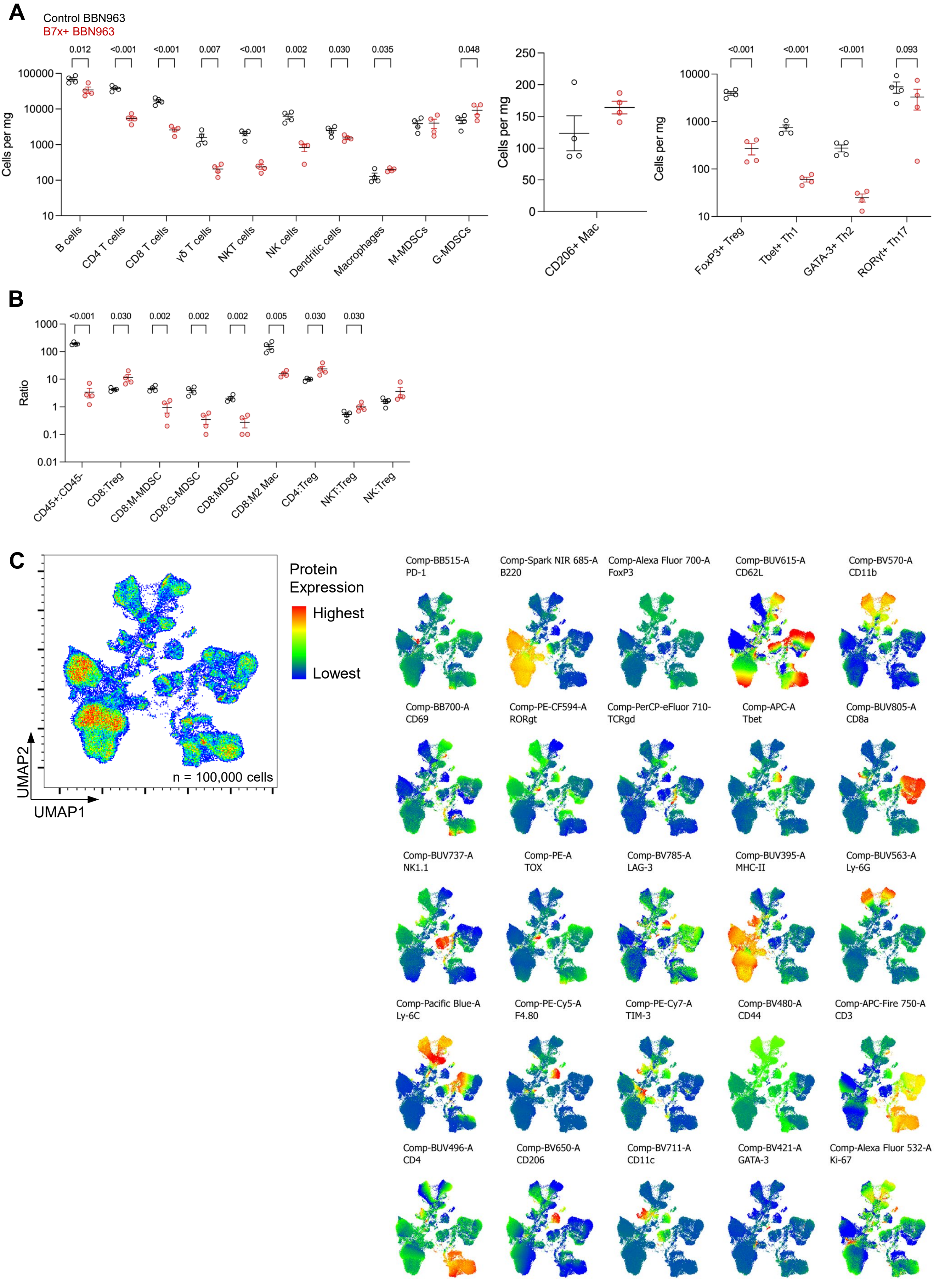

A
Control BBN963
B7x+ BBN963
0.012
<0.001
<0.001
0.007
<0.001
0.002
0.030
0.035
0.048
Cells per mg
B cells
CD4 T cells
CD8 T cells
γδ T cells
NKT cells
NK cells
Dendritic cells
Macrophages
M-MDSCs
G-MDSCs
CD206+ Mac
FoxP3+ Treg
Tbet+ Th1
GATA-3+ Th2
RORγt+ Th17
0.093
B
Ratio
CD45+:CD45-
CD8:Treg
CD8:M-MDSC
CD8:G-MDSC
CD8:MDSC
CD8:M2 Mac
CD4:Treg
NKT:Treg
NK:Treg
0.005
C
Protein Expression
Highest
Lowest
n = 100,000 cells
UMAP1
UMAP2
Comp-BB515-A PD-1
Comp-Spark NIR 685-A B220
Comp-Alexa Fluor 700-A FoxP3
Comp-BUV615-A CD62L
Comp-BV570-A CD11b
Comp-BB700-A CD69
Comp-PE-CF594-A RORgt
Comp-PerCP-eFluor 710-A TCRgd
Comp-APC-A Tbet
Comp-BUV805-A CD8a
Comp-BUV737-A NK1.1
Comp-PE-A TOX
Comp-BV785-A LAG-3
Comp-BUV395-A MHC-II
Comp-BUV563-A Ly-6G
Comp-Pacific Blue-A Ly-6C
Comp-PE-Cy5-A F4.80
Comp-PE-Cy7-A TIM-3
Comp-BV480-A CD44
Comp-APC-Fire 750-A CD3
Comp-BUV496-A CD4
Comp-BV650-A CD206
Comp-BV711-A CD11c
Comp-BV421-A GATA-3
Comp-Alexa Fluor 532-A Ki-67

Figure S4

Supplementary Fig. 4. B7x+ Bladder Tumors Alter Splenic Immune Cell Populations.

(A) Quantification of immune cell populations per mg of splenic tissue in spleens from B7x+ and B7x- BBN963 tumor-bearing mice (n = 4 spleens per group). Multiple unpaired t-tests were used (FDR q-value = 5%).

(B) Ratios of antitumor to immunosuppressive immune cell populations in spleens analyzed in panel **(A)**. Multiple unpaired t-tests were used (FDR q-value = 5%).

(C) UMAP plots illustrating protein expression of lineage and phenotypic markers in splenic immune cell populations from concatenated spleens from B7x+ and B7x- tumor-bearing mice.

Data in **(A)** and **(B)** are presented as mean ± SEM, with each dot representing a spleen. Corresponding *P* values are indicated.

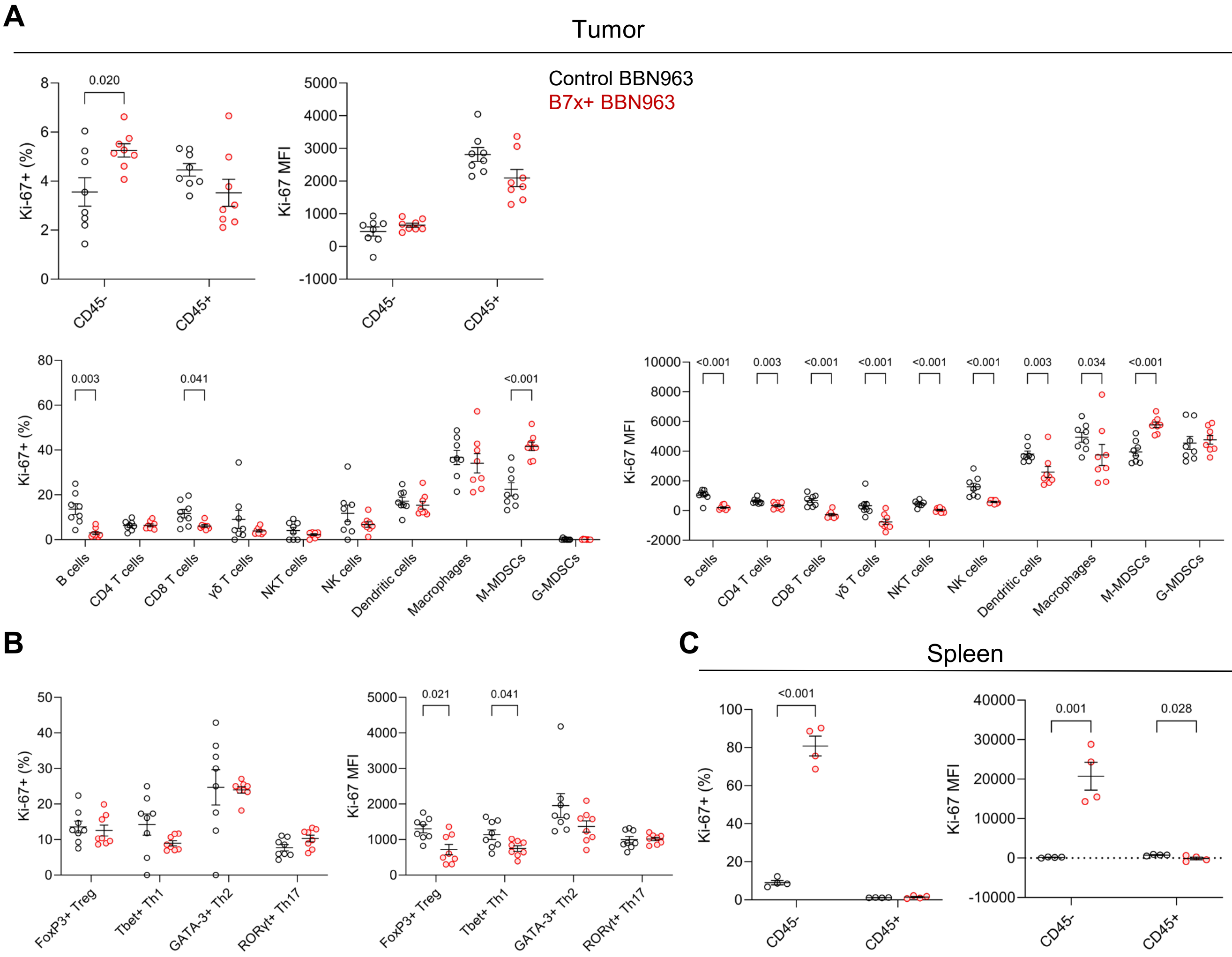
A
Tumor
Control BBN963
B7x+ BBN963
Ki-67+ (%)
Ki-67 MFI
0.020
CD45-
CD45+
0.003
0.041
<0.001
B cells
CD4 T cells
CD8 T cells
γδ T cells
NKT cells
NK cells
Dendritic cells
Macrophages
M-MDSCs
G-MDSCs
<0.001
0.003
<0.001
<0.001
<0.001
<0.001
0.003
0.034
<0.001
B
0.021
0.041
FoxP3+ Treg
Tbet+ Th1
GATA-3+ Th2
RORγt+ Th17
C
Spleen
<0.001
0.001
0.028

Figure S5

Supplementary Fig. 5. B7x+ Bladder Tumor Microenvironment Consists Primarily of Recruited Immune Cells, with Proliferating M-MDSCs.

(A-B) Protein expression of Ki-67 and the proportion of Ki-67+ tumor-infiltrating CD45- non-immune cells and CD45+ immune cells in B7x+ and B7x- BBN963 tumors (n = 8 per group). Multiple unpaired t-tests were used (FDR q-value = 5%).

(C) Protein expression of Ki-67 and the proportion of Ki-67+ $CD45^{-}$ non-immune and $CD45^{+}$ immune cells in the spleens of B7x+ and B7x- tumor-bearing mice (n = 4 per group). Multiple unpaired t-tests were used (FDR q-value = 5%).

Data in **(A-C)** are presented as mean ± SEM, with each dot representing a tumor or spleen. Corresponding *P* values are indicated.

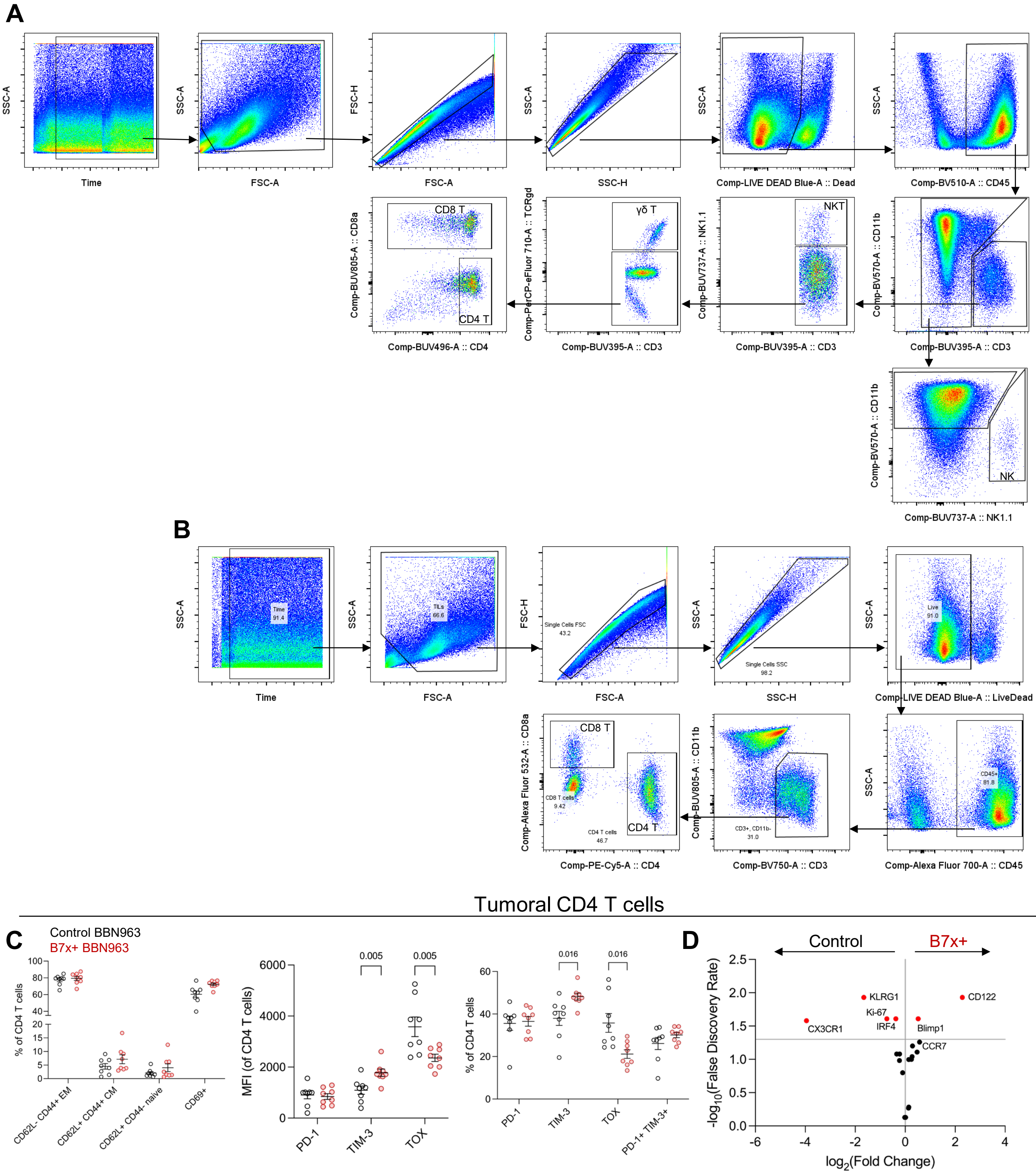
A
SSC-A
Time
FSC-A
FSC-H
SSC-H
Comp-LIVE DEAD Blue-A :: Dead
Comp-BV510-A :: CD45
CD8 T
CD4 T
Comp-BUV805-A :: CD8a
Comp-BUV496-A :: CD4
γδ T
Comp-PerCP-eFluor 710-A :: TCRgd
Comp-BUV395-A :: CD3
NKT
Comp-BUV737-A :: NK1.1
Comp-BV570-A :: CD11b
NK
B
Time 91.4
TILs 66.6
Single Cells FSC 43.2
Single Cells SSC 98.2
Live 91.0
Comp-LIVE DEAD Blue-A :: LiveDead
CD45+ 81.8
Comp-Alexa Fluor 700-A :: CD45
CD3+, CD11b- 31.0
Comp-BUV805-A :: CD11b
Comp-BV750-A :: CD3
CD8 T cells 9.42
CD4 T cells 46.7
Comp-Alexa Fluor 532-A :: CD8a
Comp-PE-Cy5-A :: CD4
Tumoral CD4 T cells
C
Control BBN963
B7x+ BBN963
% of CD4 T cells
CD62L- CD44+ EM
CD62L+ CD44+ CM
CD62L+ CD44- naive
CD69+
MFI (of CD4 T cells)
PD-1
TIM-3
TOX
0.005
0.005
PD-1+ TIM-3+
0.016
0.016
D
Control
B7x+
-log10(False Discovery Rate)
log2(Fold Change)
KLRG1
CD122
Ki-67
IRF4
CX3CR1
Blimp1
CCR7

Figure S6

Supplementary Fig. 6. B7x+ TME induces early dysfunction in antigen-experienced T cells.

(A) Gating strategy to identify CD8 and CD4 T cells, γδ T cells, NKT cells, and NK cells in B7x+ and B7x- BBN963 tumors for cytokine expression and the proportion of cytokine+ cells.

(B) Gating strategy for identifying CD8 and CD4 T cells from **(A)** to assess memory and exhaustion phenotypes.

(C) Proportion of memory or exhausted CD4 T cells, along with expression of memory or exhaustion markers, in B7x+ and B7x- tumors (n = 8 tumors per group). Data are presented as mean ± SEM, with each dot representing a tumor. Multiple unpaired t-tests were used (FDR q-value = 5%).

(D) Volcano plot depicting the fold-change in protein expression for each memory marker in CD4 T cells.

Corresponding *P* values are indicated.

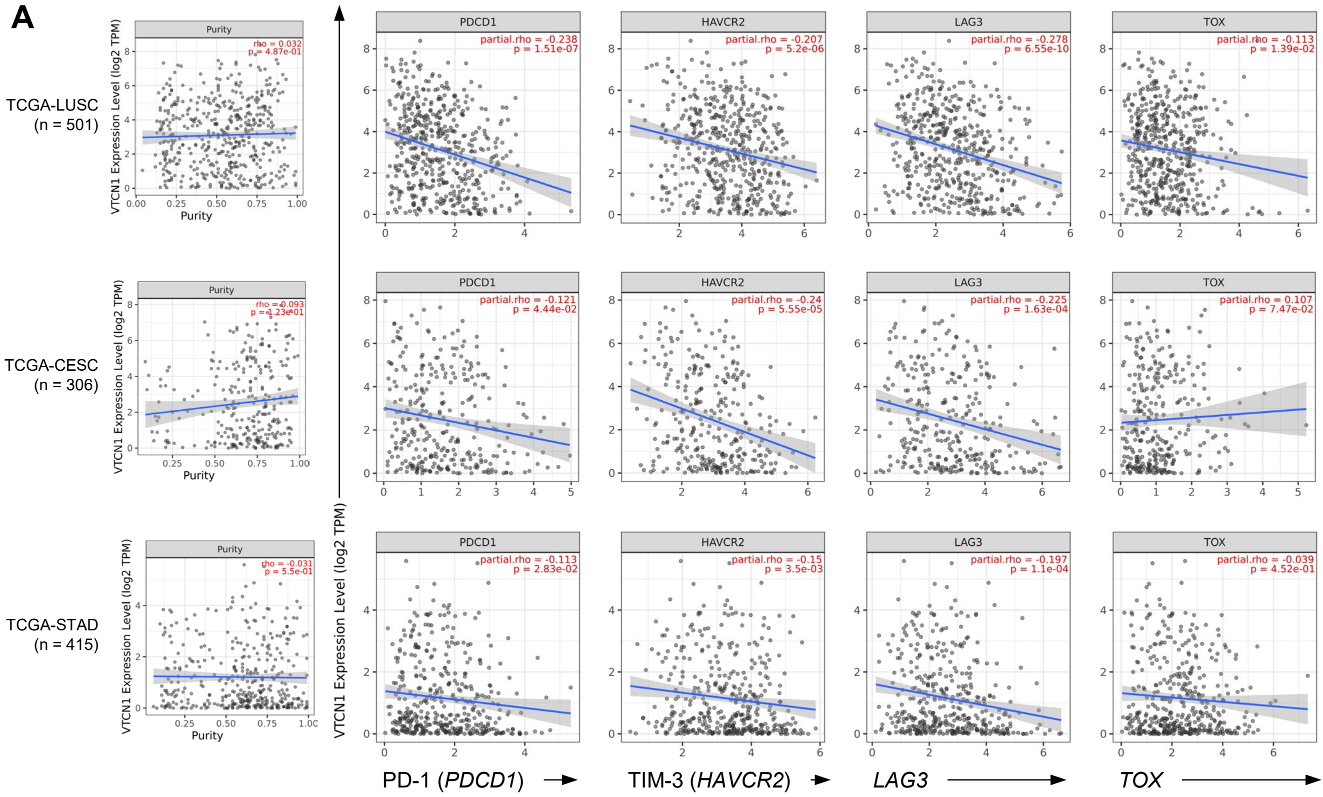
A
TCGA-LUSC (n = 501)
TCGA-CESC (n = 306)
TCGA-STAD (n = 415)
VTCN1 Expression Level (log2 TPM)
Purity
PDCD1
HAVCR2
LAG3
TOX
partial.rho = -0.238 p = 1.51e-07
partial.rho = -0.207 p = 5.2e-06
partial.rho = -0.278 p = 6.55e-10
partial.rho = -0.113 p = 1.39e-02
partial.rho = -0.121 p = 4.44e-02
partial.rho = -0.24 p = 5.55e-05
partial.rho = -0.225 p = 1.63e-04
partial.rho = 0.107 p = 7.47e-02
partial.rho = -0.113 p = 2.83e-02
partial.rho = -0.15 p = 3.5e-03
partial.rho = -0.197 p = 1.1e-04
partial.rho = -0.039 p = 4.52e-01
PD-1 (PDCD1)
TIM-3 (HAVCR2)
LAG3
TOX

Figure S7

Supplementary Fig. 7. B7x mRNA expression negatively correlates with exhaustion markers in some human cancers.

(A) Correlations between the mRNA expression of B7x and exhaustion markers (PD-1, LAG3, TIM-3, and TOX) were analyzed in Lung Squamous Cell Carcinoma (LUSC), Cervical Squamous Cell Carcinoma and Endocervical Adenocarcinoma (CESC), and Stomach Adenocarcinoma (STAD) using RNA-seq data from the TCGA database. Best-fit linear regression lines are shown in blue, with the 95% confidence interval displayed in grey, and the purity-adjusted Spearman's Rho and *P* values indicated in red.

A

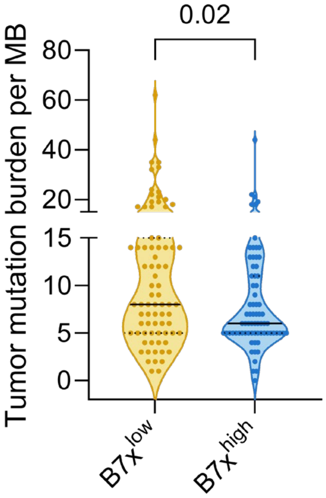

0.02
Tumor mutation burden per MB
80
60
40
20
15
10
5
0
B7x low
B7x high

Figure S8

Supplementary Fig. 8. High B7x expression correlates with lower tumor mutation burden in PD-L1 blockade-treated MIBC patients.

(A) Violin plot comparing tumor mutation burden per megabase in MIBC patients from the IMvigor210 clinical trial, stratified by B7x mRNA expression. Each dot represents an individual patient. Mann-Whitney U test was used. Corresponding *P* value is indicated.

Supplementary Table S1. Antibodies Used for Flow Cytometry

Supplementary Table S2. Demographic and Clinical Characteristics of the TCGA-BLCA Patients.

Supplementary Table S3. Demographic and Clinical Characteristics of the IMvigor210 Patients.

Supplementary Table S1A. Antibodies Used for Comprehensive Immunophenotyping

Target Protein	Clone	Fluorophore	Dilution	Manufacturer	Identifier (Cat #)
I-A/I-E (MHC-II)	2G9	BUV395	1:200	BD Biosciences	743876
CD4	GK1.5	BUV496	1:300	BD Biosciences	612952
Ly6G	1A8	BUV563	1:200	BD Biosciences	612921
CD62L	MEL-14	BUV615	1:300 - 400	BD Biosciences	752311
NK1.1	PK136	BUV737	1:100	BD Biosciences	741715
CD8a	53-6.7	BUV805	1:200	BD Biosciences	612898
GATA-3	L50-823	BV421	1:200	BD Biosciences	563349
Ly6C	HK1.4	Pacific Blue	1:500	BioLegend	128013
CD44	IM7	BV480	1:400	BD Biosciences	566200
CD45	30-F11	BV510	1:400	BD Biosciences	563891
CD11b	M1/70	BV570	1:400	BioLegend	101233
CD206	C068C2	BV650	1:500 - 600	BioLegend	141723
CD11c	N418	BV711	1:500	BioLegend	117349
LAG-3	C9B7W	BV785	1:100	BioLegend	125219
PD-1	RMP1-30	BB515	1:200	BD Biosciences	566832
Ki-67	SolA15	Alexa Fluor 532	1:800	eBioscience	58-5698-82
CD69	H1.2F3	BB700	1:100	BD Biosciences	566500
TCR γ/δ	GL3	PerCp-eFluor 710	1:100	eBioscience	46-5711-82
TOX	TXRX10	PE	1:100	eBioscience	12-6502-82
RORyt	Q31-378	PE-CF594	1:300 - 400	BD Biosciences	562684
F4/80	BM8	PE-Cy5	1:400 - 500	BioLegend	123111
TIM-3	RMT3-23	PE-Cy7	1:100	BioLegend	119715
Tbet	4B10	APC	1:100	BioLegend	644813
B220	RA3-6B2	Spark NIR 685	1:300	BioLegend	103267
Foxp3	FJK-16s	AF700	1:200	eBioscience	56-5773-82
CD3	17A2	APC/Fire750	1:100	BioLegend	100247

Supplementary Table S1B. Antibodies Used for Cytokine immunophenotyping

Target Protein	Clone	Fluorophore	Dilution	Manufacturer	Identifier (Cat #)
CD3	17A2	BUV395	1:100	BD Biosciences	569614
CD4	GK1.5	BUV496	1:600	BD Biosciences	612952
NK1.1	PK136	BUV737	1:100	BD Biosciences	741715
CD8a	53-6.7	BUV805	1:200	BD Biosciences	612898
CD45	30-F11	BV510	1:200	BD Biosciences	563891
CD11b	M1/70	BV570	1:400	BioLegend	101233
TNFα	MP6-XT22	BV785	1:1000	BioLegend	506341
IL-17A	TC11-18H10.1	FITC	1:400	BioLegend	506907
TCR γ/δ	GL3	PerCp-eFluor 710	1:100	eBioscience	46-5711-82
IL-2	JES6-5H4	PE	1:200	BD Biosciences	561061
IFNγ	XMG1.2	PE-CF594	1:1600	BD Biosciences	562333
Granzyme B	NGZB	PE-Cy7	1:600	eBioscience	25-8898-82
IL-4	11B11	APC	1:200	BioLegend	504105
CD107a	1D4B	APC-Cy7	1:800	BioLegend	121615

Supplementary Table S1C. Antibodies Used for T cell Memory immunophenotyping

Target Protein	Clone	Fluorophore	Dilution	Manufacturer	Identifier (Cat #)
Sca-1	D7	BUV395	1:400	BD Biosciences	563990
CD122	TM-β1	BUV496	1:100	BD Biosciences	741073
PD-1	RMP1-30	BUV737	1:100	BD Biosciences	568363
CD11b	M1/70	BUV805	1:400	BD Biosciences	568345
CD127	A7R34	BV421	1:200	BD Biosciences	566377
CD44	IM7	Super Bright 436	1:200	eBioscience	62-0441-82
Bcl-2	10C4	eFluor450	1:400	eBioscience	48-6992-42
Ki-67	B56	BV480	1:100	BD Biosciences	566109
CX3CR1	SA011F11	BV510	1:200	BioLegend	149025
CD62L	MEL-14	BV570	1:200	BioLegend	104433
KLRG1	2F1/KLRG1	BV605	1:200	BioLegend	138419
CD27	LG.3A10	BV650	1:200	BioLegend	124233
Tbet	4B10	BV711	1:100	BioLegend	644819
CD3	17A2	BV750	1:200	BioLegend	100249
CCR7	4B12	BV786	1:50	BD Biosciences	564355
CD95	Jo2	BB515	1:200	BD Biosciences	565605
TCF1	S33-966	Alexa Fluor 488	1:200	BD Biosciences	567018
CD8a	53-6.7	Alexa Fluor 532	1:400	eBioscience	58-0081-80
CXCR3	CXCR3-173	BB700	1:200	BD Biosciences	742274
CCR5	HM-CCR5 (7A4)	PerCp-eFluor 710	1:50	eBioscience	46-1951-82
CD4	GK1.5	PE-Cy5	1:300	BioLegend	100409
IRF4	IRF4.3E4	PE-Cy7	1:900	BioLegend	646413
Blimp1	5E7	APC	1:100	BioLegend	150007
EOMES	Dan11mag	eFluor 660	1:50	eBioscience	50-4875-82
CD45	30-F11	AF700	1:300	BioLegend	103127
Bcl-6	K112-91	APC-Cy7	1:200	BD Biosciences	563581

Supplementary Table S2A. Demographic and Clinical Characteristics of the TCGA-BLCA Patients (Full Analysis Population).

Characteristic	B7xlow (N =102)	B7xhigh (N=101)	Total (N=203)	P-value	Test
**Age**				0.77	Fisher's Exact
<=65 years	36 (48.6%)	38 (51.4%)	74		
>65 years	66 (51.2%)	63 (48.8%)	129		
**Gender**				0.63	Fisher's Exact
Female	28 (53.8%)	24 (46.2%)	52		
Male	74 (49.0%)	77 (51.0%)	151		
**Race**				0.17	Chi-squared
African American	4 (36.4%)	7 (63.6%)	11		
Asian	13 (68.4%)	6 (31.6%)	19		
White	78 (48.4%)	83 (51.6%)	161		
Not Documented (ND)	7 (58.3%)	5 (41.7%)	12		
**Tobacco_smoking_history**				0.54	Chi-squared
Current reformed smoker for < or = 15 years	22 (56.4%)	17 (43.6%)	39		
Current reformed smoker for > 15 years	25 (44.6%)	31 (55.4%)	56		
Current reformed smoker, duration not specified	3 (33.3%)	6 (66.7%)	9		
Current smoker	25 (56.8%)	19 (43.2%)	44		
Lifelong non-smoker	24 (49.0%)	25 (51.0%)	49		
ND	3 (50.0%)	3 (50.0%)	6		
**Histologic_subtype**				0.22	Fisher's Exact
Non-papillary	66 (47.5%)	73 (52.5%)	139		
Papillary	34 (57.6%)	25 (42.4%)	59		
ND	2 (40.0%)	3 (60.0%)	5		
**Molecular subtype (mRNA_cluster)**				0.00010	Chi-squared
Basal_squamous	54 (68.4%)	25 (31.6%)	79		
Luminal	2 (16.7%)	10 (83.3%)	12		
Luminal_infiltrated	11 (25.0%)	33 (75.0%)	44		
Luminal_papillary	31 (50.8%)	30 (49.2%)	61		
Neuronal	4 (57.1%)	3 (42.9%)	7		
**TNM_tumor_stage**				0.19	Chi-squared
T1	0 (0.0%)	1 (100.0%)	1		
T2	37 (59.7%)	25 (40.3%)	62		
T3	47 (51.1%)	45 (48.9%)	92		
T3b	0 (0.0%)	1 (100.0%)	1		
T4	12 (40.0%)	18 (60.0%)	30		
T4a	0 (0.0%)	1 (100.0%)	1		
ND	6 (37.5%)	10 (62.5%)	16		
**TNM_lymphovascular_invasion**				0.40	Fisher's Exact
No	27 (42.9%)	36 (57.1%)	63		
Yes	40 (50.6%)	39 (49.4%)	79		
ND	35 (57.4%)	26 (42.6%)	61		
**TNM_lymph_node_stage**				0.40	Chi-squared
N0	60 (55.0%)	49 (45.0%)	109		
N1	14 (51.9%)	13 (48.1%)	27		
N2	15 (39.5%)	23 (60.5%)	38		
N3	2 (66.7%)	1 (33.3%)	3		
NX	9 (39.1%)	14 (60.9%)	23		
**TNM_metastatic_stage**				0.19	Chi-squared
M0	48 (58.5%)	34 (41.5%)	82		
M1	3 (42.9%)	4 (57.1%)	7		
MX	51 (45.1%)	62 (54.9%)	113		
**Stage**				0.55	Chi-squared
I	0 (0.0%)	1 (100.0%)	1		
II	36 (56.3%)	28 (43.8%)	64		
III	33 (49.3%)	34 (50.7%)	67		
IV	33 (47.1%)	37 (52.9%)	70		
ND	0 (0.0%)	1 (100.0%)	1		
**Grade**				0.014	Fisher's Exact
Low Grade	7 (100.0%)	0 (0.0%)	7		
High Grade	94 (48.7%)	99 (51.3%)	193		
ND	1 (33.3%)	2 (66.7%)	3		
**Presence_of_prostate_cancer**				0.86	Fisher's Exact
No	58 (49.6%)	59 (50.4%)	117		
Yes	21 (46.7%)	24 (53.3%)	45		
ND	22 (56.4%)	17 (43.6%)	39		
Not Available	1 (50.0%)	1 (50.0%)	2		
**Stage_of_prostate_cancer**				0.19	Chi-squared
pT1a	2 (50.0%)	2 (50.0%)	4		
pT2	2 (100.0%)	0 (0.0%)	2		
pT2a	6 (33.3%)	12 (66.7%)	18		
pT2b	2 (40.0%)	3 (60.0%)	5		
pT2c	5 (62.5%)	3 (37.5%)	8		
pT3	1 (100.0%)	0 (0.0%)	1		
pT3a	0 (0.0%)	2 (100.0%)	2		
pT3b	2 (100.0%)	0 (0.0%)	2		
ND	80 (50.6%)	78 (49.4%)	158		
Not Available	2 (66.7%)	1 (33.3%)	3		
**Squamous_pathology**				0.26	Fisher's Exact
No	88 (48.6%)	93 (51.4%)	181		
Yes	14 (63.6%)	8 (36.4%)	22		
**Neuroendocrine_pathology**				1.00	Fisher's Exact
No	102 (50.2%)	101 (49.8%)	203		
Yes	0	0	0		
**Plasmacytoid_pathology**				0.25	Fisher's Exact
No	102 (50.7%)	99 (49.3%)	201		
Yes	0 (0.0%)	2 (100.0%)	2		

Supplementary Table S2B. Mutation process cluster, APOBEC mutation load, and Neoantigen load of the TCGA-BLCA Patients (Full Analysis Population).

Characteristic	B7xlow (N =102)	B7xhigh (N=101)	Total (N=203)	P-value	Test
**Mutation_process_cluster**				1.00	Chi-squared
Msig_1	4 (57.1%)	3 (42.8%)	7		
Msig_2	53 (49.5%)	54 (50.5%)	107		
Msig_3	28 (50.9%)	27 (40.1%)	55		
Msig_4	17 (50%)	17 (50%)	34		
**APOBEC_mutation_load**				0.24	Chi-squared
No	15 (65.2%)	8 (34.8%)	23		
Low	41 (45.6%)	49 (54.4%)	90		
High	46 (51.1%)	44 (48.9%)	90		
**Neoantigen_load**				0.43	Chi-squared
1st_quartile	27 (60%)	18 (40%)	45		
2nd_quartile	28 (49.1%)	29 (50.9%)	57		
3rd_quartile	22 (43.1%)	29 (56.9%)	51		
4th_quartile	25 (50%)	25 (50%)	50		

Supplementary Table S3A. Demographic and Clinical Characteristics of the IMvigor210 Patients (Full Analysis Population).

Characteristic	B7xlow (N =96)	B7xhigh (N=96)	Total (N=192)	P-value	Test
**Response**				0.030	Fisher's Exact
Complete Response (CR)	18 (72.0%)	7 (28.0%)	25		
Progressive Disease (PD)	78 (46.7%)	89 (53.3%)	167		
**Lund**				0.005	Chi-squared
Urobasal A (UroA)	30 (50.0%)	30 (50.0%)	60		
Urobasal B (UroB)	7 (63.6%)	4 (36.4%)	11		
Genomically Unstable (GU)	6 (24.0%)	19 (76.0%)	25		
Squamous Cell Carcinoma-Like (SCCL)	31 (68.9%)	14 (31.1%)	45		
Infiltrated (Inf)	22 (43.1%)	29 (56.9%)	51		
**TCGA Subtype**				0.009	Chi-squared
I	38 (55.1%)	31 (44.9%)	69		
II	13 (28.3%)	33 (71.7%)	46		
III	25 (58.1%)	18 (41.9%)	43		
IV	20 (58.8%)	14 (41.2%)	34		
**Immune Cells (IC)**				0.023	Chi-squared
IC0	25 (44.6%)	31 (55.4%)	56		
IC1	31 (42.5%)	42 (57.5%)	73		
IC2+	40 (64.5%)	22 (35.5%)	62		
N/A	0 (0.0%)	1 (100.0%)	1		
**Tumor Cells (TC)**				0.406	Chi-squared
TC0	80 (52.3%)	73 (47.7%)	153		
TC1	4 (33.3%)	8 (66.7%)	12		
TC2+	12 (46.2%)	14 (53.8%)	26		
N/A	0 (0.0%)	1 (100.0%)	1		
**TP53**				0.874	Fisher's Exact
Mutant	39 (51.3%)	37 (48.7%)	76		
Non-mutant	39 (50.0%)	39 (50.0%)	78		
N/A	18 (47.4%)	20 (52.6%)	38		
**RB1**				1.000	Fisher's Exact
Mutant	10 (52.6%)	9 (47.4%)	19		
Non-mutant	68 (50.4%)	67 (49.6%)	135		
N/A	18 (47.4%)	20 (52.6%)	38		
**FGFR3**				0.039	Fisher's Exact
Mutant	20 (69.0%)	9 (31.0%)	29		
Non-mutant	58 (46.4%)	67 (53.6%)	125		
N/A	18 (47.4%)	20 (52.6%)	38		
**CDKN2A**				0.842	Fisher's Exact
Mutant	15 (48.4%)	16 (51.6%)	31		
Non-mutant	63 (51.2%)	60 (48.8%)	123		
N/A	18 (47.4%)	20 (52.6%)	38		
**ERBB2**				0.015	Fisher's Exact
Mutant	3 (20.0%)	12 (80.0%)	15		
Non-mutant	75 (54.0%)	64 (46.0%)	139		
N/A	18 (47.4%)	20 (52.6%)	38		
**PIK3CA**				1.000	Fisher's Exact
Mutant	12 (50.0%)	12 (50.0%)	24		
Non-mutant	66 (50.8%)	64 (49.2%)	130		
N/A	18 (47.4%)	20 (52.6%)	38		

Supplementary Table S3B. PD-L1 protein expression in IMvigor210 bladder cancer specimens (%)

B7x Expression (Low/High)	Immune Cell (IC)_Level	Immune Cell (IC)_count	Low/High_count	IC_ratio	IC_percentage_	IC_percentage	B7x_IC
Low	IC0	25	96	0.260416667	26.04166667	26.04	Low_IC0
Low	IC1	31	96	0.322916667	32.29166667	32.29	Low_IC1
Low	IC2+	40	96	0.416666667	41.66666667	41.67	Low_IC2+
High	IC0	31	95	0.326315789	32.63157895	32.63	High_IC0
High	IC1	42	95	0.442105263	44.21052632	44.21	High_IC1
High	IC2+	22	95	0.231578947	23.15789474	23.16	High_IC2+

B7x Expression (Low/High)	Tumor Cell (TC)_Level	Tumor Cell (TC)_count	Low/High_count	TC_ratio	TC_percentage_	TC_percentage	B7x_TC
Low	TC0	80	96	0.833333333	83.33333333	83.33	Low_TC0
Low	TC1	4	96	0.041666667	4.166666667	4.17	Low_TC1
Low	TC2+	12	96	0.125	12.5	12.5	Low_TC2+
High	TC0	73	95	0.768421053	76.84210526	76.84	High_TC0
High	TC1	8	95	0.084210526	8.421052632	8.42	High_TC1
High	TC2+	14	95	0.147368421	14.73684211	14.74	High_TC2+

Test	PD-L1 expression	Chi-squared	Pval	Df
Pearson's Chi-squared	IC	7.521168406	0.023270142	2
Pearson's Chi-squared	TC	1.802254726	0.406111567	2

Supplementary Table S3C. ssGSEA Scores in Bladder Cancer Specimens from IMvigor210 B7x High vs. B7x Low cohort

Group	P value	q value	Mean rank of B7xlow	Mean rank of B7xhigh	Mean rank diff.	Mann-Whitney U	log2(Fold change of B7x high/low mean rank)	negative log10(p-value)	negative log10(FDR q-value)
CD_8_T_effector	0.078	0.128	104	89.4	14.1	3930	-0.218236792	1.107905397	0.868964995
DDR	0.019	0.09	106	87.1	18.8	3708	-0.283319641	1.721246399	1.016158523
APM	0.027	0.09	105	87.7	17.7	3760	-0.25974058	1.568636236	1.016158523
Immune_Checkpoint	0.133	0.18	103	90.5	12.1	4029	-0.18665464	0.876148359	0.721437185
CC_Reg	0.317	0.316	101	92.5	8.04	4222	-0.126830022	0.498940738	0.477101888
Fanconi	0.078	0.128	104	89.4	14.1	3929	-0.218236792	1.107905397	0.868964995
Pan_F_TBRS	0.221	0.246	91.6	101	-9.83	4136	0.14093579	0.655607726	0.585624429
tcga	0.483	0.457	93.7	99.3	-5.65	4337	0.08374467	0.316052869	0.316678052
Histones	0.072	0.128	104	89.3	14.4	3916	-0.219851448	1.142667504	0.868964995
EMT1	3.25915E-05	<0.001	79.8	113	-33.3	3008	0.501862121	4.486895855	3.185865859
EMT2	0.262	0.275	92	101	-9.02	4175	0.134649527	0.581698709	0.537143049
EMT3	0.189	0.223	91.2	102	-10.6	4101	0.161463423	0.723538196	0.628211814
WNT_target	0.002	0.015	109	83.9	25.3	3395	-0.377585419	2.698970004	1.786142974
FGFR3_related	0.169	0.213	102	91	11	4078	-0.164630702	0.772113295	0.647099085
Cell_cycle	0.032	0.09	105	87.9	17.1	3785	-0.256454257	1.494850022	1.016158523
Mismatch_Repair	0.112	0.162	103	90.1	12.8	3995	-0.193045326	0.950781977	0.76513699
Homologous_recombination	0.079	0.128	104	89.5	14.1	3932	-0.216623941	1.102372909	0.868964995
Nucleotide_excision_repair	0.034	0.09	105	88	17	3790	-0.254813899	1.468521083	1.016158523
DNA_replication	0.081	0.128	104	89.5	14	3936	-0.216623941	1.091514981	0.868964995
Base_excision_repair	0.017	0.09	106	86.9	19.1	3690	-0.286636183	1.769551079	1.016158523
